# A histopathology image–based computer-aided classification study for oral squamous cell carcinoma

**DOI:** 10.3389/fonc.2026.1806332

**Published:** 2026-04-14

**Authors:** Yiping Ren, Runwen Li, Dong Chen

**Affiliations:** 1 Department of Stomatology, Panzhihua Central Hospital, Panzhihua, Sichuan, China; 2 Department of Vascular Diseases, Panzhihua Central Hospital, Panzhihua, China; 3 Department of Obstetrics, Panzhihua Central Hospital, Panzhihua, Sichuan, China

**Keywords:** histopathological image classification, multiple-instance learning, oral squamous cell carcinoma, staining-bias suppression, structured aggregation.

## Abstract

**Introduction:**

Oral squamous cell carcinoma histopathological image classification is often challenged by staining variations and sparse local lesions, which can cause models to overfit color differences and weaken cross-domain generalization.

**Methods:**

A classification framework combining staining-bias suppression and structured multiple-instance aggregation was developed. In representation learning, stain-related features were disentangled from morphological and structural information, and a gated suppression mechanism was introduced to reduce color interference while enhancing tissue architecture and cellular morphology cues. In decision aggregation, image patches were treated as instances and spatial priors were incorporated to capture both neighborhood continuity and long-range dependencies.

**Results:**

The proposed method achieved Acc 87.35%, F1 91.27%, and AUC 98.04% on one test set, and Acc 79.34%, F1 86.86%, and AUC 90.74% on another test set. It consistently outperformed traditional and deep learning baselines. External validation on an independent retrospective clinical cohort from a local hospital also showed stable performance.

**Discussion:**

The results indicate that the proposed method can effectively alleviate the impact of staining bias and improve classification robustness. Its strong performance on external data further supports its practical value under real-world acquisition and staining variations.

## 1 Introduction

Oral squamous cell carcinoma (OSCC) is one of the most common pathological types of oral malignancies, and its early detection and accurate diagnosis are of critical clinical importance for reducing recurrence risk and improving survival rates. Histopathological examination is the gold standard for oral cancer diagnosis; however, in real-world workflows, slide reading relies heavily on experienced pathologists, which is time-consuming and subject to inter-observer variability Tan et al. (1). This issue becomes more prominent in large-scale screening scenarios and in settings with limited primary healthcare resources. With the development of digital pathology and deep learning, intelligent computer-aided diagnosis based on histopathology images has become a feasible direction Speight et al. (2)Zhou and Lu (3). In particular, automatic recognition based on the binary classification task between Normal and OSCC can provide effective support for subsequent grading assessment and clinical decision-making. Therefore, developing oral cancer histopathology image classification methods with high accuracy, strong robustness, and good interpretability has significant research value and practical significance.

Although existing methods have achieved progress on specific datasets, oral histopathology images often exhibit substantial distribution shifts across centers, devices, and staining batches, making models vulnerable to staining style differences and changes in imaging conditions and consequently suffering from degraded generalization performance Van der Laak et al. (4)Zhou and Lu (5). In particular, color bias introduced by H&E staining may induce networks to learn diagnosis-irrelevant color-driven spurious correlations, leading to unstable behavior when transferring across domains Tellez et al. (6)Gadermayr and Tschuchnig (7). On the other hand, discriminative cues in histopathology images are typically localized, and there exist explicit spatial topological relations among different patches. If only simple global pooling or independent patch-wise prediction with voting is adopted, the structural consistency and spatial dependencies among local evidences can be overlooked, weakening the ability to represent complex tissue architectures. These issues jointly hinder the reliable deployment of oral histopathology image classification models in real clinical scenarios.

To address these challenges, we propose a structured learning framework for OSCC histopathology image classification, which performs explicit robustness enhancement and structured aggregation on mid-to-high-level features extracted by the backbone network. First, we introduce a morphology-dominant encoding module with staining-bias suppression after the backbone features. This module performs factorized modeling by decomposing features into stain-related and structure-related components, and employs a gating mechanism to suppress color-sensitive components while strengthening morphology-structural components, thereby making the representation focus more on diagnostically stable morphological cues. Second, we design a Spatial Transformer Classifier Head that explicitly models spatial topological relations at the patch level and performs structured aggregation, thereby leveraging neighborhood consistency and non-local dependencies to improve overall decision stability and interpretability while preserving local discriminative capability. Based on an experimental setup on two public datasets, we systematically validate the proposed method through conventional comparisons, ablation studies, visualization-based explanations, and cross-dataset transfer evaluations.

The main contributions of this work are summarized as follows:

1. We propose a morphology-dominant encoding strategy with staining-bias suppression, which performs feature decomposition and gated reweighting on backbone outputs to reduce the interference of staining style differences on representation learning and enhance morphology robust representations.
2. We design a Spatial Transformer Classifier Head that explicitly models spatial topological relations among patch features and performs structured aggregation, improving the integration of local pathological evidence and the modeling of cross-region consistency.
3. We conduct comprehensive evaluations on two public oral histopathology image datasets, including comparisons with multiple baseline models, ablation studies, hyperparameter sensitivity analysis, and interpretability visualizations, and further perform cross-dataset transfer experiments to validate both the robustness and limitations of the proposed method under distribution shifts.

## 2 Related work

### 2.1 Oral cancer histopathological image classification and multiple-instance learning

In recent years, intelligent classification of histopathology images for oral squamous cell carcinoma (OSCC) has developed rapidly. Mainstream studies are mostly built on convolutional networks or transfer learning, and perform supervised learning directly on whole-slide images or local regions. Yang et al. Yang et al. (8) demonstrated the feasibility of deep learning for OSCC pathological diagnosis and its potential clinical value; Sukegawa et al. Sukegawa et al. (9) further validated the effectiveness of deep classifiers in real diagnostic workflows from the perspective of collaborative evaluation by pathologists. Meanwhile, Panigrahi et al. Panigrahi et al. (10) and Ahmad et al. Ahmad et al. (11) improved recognition performance under different data conditions through transfer learning and hybrid feature strategies. Das et al. Das et al. (12) showed that deep models can capture microscopic morphological details at the level of multi-class cellular categories, and Das et al. Das et al. (13) also provided an end-to-end detection practice for oral mucosal histopathology images. These methods share clear implementation pathways and are easy to train and deploy, but they also have notable limitations. First, pathological images exhibit strong spatial heterogeneity, and decisive lesions often occupy only local regions; direct whole-image classification or independent patch classification can be easily disturbed by non-critical areas, leading to unstable decisions. Second, distribution shifts introduced by different slides and experimental procedures can induce the model to learn shortcut features, thereby weakening cross-source generalization.

To alleviate these issues, weakly supervised multiple instance learning (MIL) has gradually become an important paradigm for pathology image classification. It achieves image-level prediction by aggregating a set of patches and naturally fits clinical scenarios with scarce annotations. Koriakina et al. Koriakina et al. (14) systematically compared deep MIL and conventional single-instance learning for interpretable oral cancer detection, indicating that MIL has advantages in focusing on key regions and improving interpretability. In addition, Zhou et al. Zhou et al. (15) introduced semi-supervised learning into OSCC diagnosis and prognosis modeling, reflecting the trend of improving robustness under limited annotations. Lian et al. Lian et al. (16) explored the feasibility of AI detection under more complex imaging conditions such as cytological autofluorescence, suggesting that domain discrepancies in real-world settings remain a central challenge. Although MIL can highlight highly contributive instances via attention or aggregation mechanisms, existing methods often under-model the spatial topological relations among patches, and the aggregation weights may exhibit discrete and incoherent responses, which weakens the characterization and explanation of tissue structural patterns such as tumor-nest clusters and invasion boundaries. Recent studies have begun to explicitly exploit spatial dependencies to regularize or enhance WSI-level aggregation. Wu et al. Wu et al. (17) proposed a label-independent regularization strategy that leverages spatial patterns among patches as an auxiliary constraint, improving the coherence of instance responses without requiring dense annotations. In parallel, graph-based MIL frameworks model patches as nodes and encode their neighborhood relations to capture tissue topology beyond independent instance voting. In particular, Zheng et al. Zheng et al. (18) introduced a graph-transformer architecture for whole-slide classification to learn long-range contextual interactions on a constructed patch graph, while Ding et al. Ding et al. (19) further improved efficiency and representation capacity via multi-scale graph-transformer modeling. Moreover, cross-laboratory staining variations and imaging biases can still accumulate at the instance-feature level and propagate to the aggregated decision. Compared with these spatial-dependency regularization and graph-aggregation paradigms, our structured aggregation module is designed to impose explicit spatial topological constraints during fusion, and our morphology-driven representation learning targets staining-induced shortcut features at the instance level, thereby jointly improving cross-domain stability and structural consistency. To address these limitations, we plan to introduce morphology-driven robust representations and a structured aggregation mechanism with spatial topological constraints into the MIL framework, so as to emphasize key lesion regions while improving cross-domain stability and structural consistency.

### 2.2 Stain bias and morphology-robust representation learning

In digital pathology, H&E staining is strongly dependent on laboratory procedures, reagent batches, scanners, and illumination settings, which leads to substantial staining variation and inter-domain distribution shifts. As a result, deep models can easily learn color-based shortcut features that are unrelated to the disease, thereby weakening cross-center generalization. To address this issue, early studies mainly followed classical pipelines of stain normalization and stain separation. Khan et al. Khan et al. (20) achieved color standardization via image-specific color deconvolution and nonlinear mapping. Vahadane et al. Vahadane et al. (21) proposed structure-preserving color normalization and combined it with sparse stain separation to preserve tissue morphology as much as possible. Janowczyk et al. Janowczyk et al. (22) introduced a sparse autoencoder for stain normalization (StaNoSA) to improve adaptability. These methods are effective in reducing color discrepancies and improving training stability, but they also have limitations. First, preprocessing often depends on reference templates or parameter settings; under extreme staining or exposure conditions, it may over-correct or introduce artifacts, which can in turn damage morphological information. Second, unified color standardization does not guarantee that the model truly focuses on nuclear morphology, boundaries, and tissue architecture; domain bias may still remain in the learned features, leading to performance fluctuations across sources. In addition to staining variation, practical WSI pipelines may also suffer from acquisition-quality factors such as defocus and blur, which can further distort local morphological cues and aggravate cross-source inconsistency; recent work has explored focus quality assessment driven deblurring to improve the reliability of digital pathology images for downstream analysis Zhou et al. (23).

To further improve robustness, subsequent work began to mitigate staining bias from the perspectives of learning strategies and representation. On the one hand, BenTaieb et al. BenTaieb and Hamarneh (24) used adversarial stain transfer to generate samples with diverse staining styles during training, enhancing the model’s tolerance to color variations. On the other hand, domain-invariant representation learning has been adopted to directly reduce discrepancies among different sources. Lafarge et al. Lafarge et al. (25) learned domain-invariant representations for histology images to improve cross-domain generalization. Anghel et al. Anghel et al. (26) constructed a robust stain normalization pipeline for whole slide images from a systems perspective to improve consistency in large-scale processing. Otálora et al. Otálora et al. (27) emphasized learning stain-invariant features to enhance generalization in computational pathology. Meanwhile, adaptive standardization algorithms such as SCAN Salvi et al. (28) and fast normalization networks such as StainNet Kang et al. (29) improved efficiency and usability. Voon et al. Voon et al. (30) also empirically evaluated the effectiveness of different stain normalization techniques for automated grading. Beyond color-oriented normalization and invariant learning, weakly supervised WSI classification has also progressed toward more robust aggregation mechanisms, where iterative MIL Zhou et al. (31) and critical-instance based MIL Zhou and Lu (32) aim to refine instance selection and stabilize slide-level decisions under noisy and heterogeneous patch distributions. However, existing methods still face two key challenges. First, strategies that rely heavily on color alignment may obscure genuine tissue differences, and the downstream gains brought by different normalization or transfer approaches are not consistently stable. Second, even when staining effects are weakened, the model still needs to explicitly strengthen morphological cues in the feature space; otherwise, unreliable decisions may occur under complex background textures and local heterogeneity. To address these limitations, we plan to introduce a factorized representation at the feature level that separates stain-related factors from morphology-dominant factors, and to employ adaptive gating to suppress staining bias and emphasize stable structural and morphological representations, thereby improving robust classification across centers and imaging conditions.

## 3 Method

### 3.1 Dataset introduction

We conduct binary classification experiments between Normal and OSCC on two publicly available oral cancer histopathology image datasets to validate the effectiveness and robustness of the proposed method under different acquisition conditions and dataset scales. Dataset 1 is collected from the oral cancer histopathology image repository released by Rahman et al. Rahman et al. (33), which contains H&E-stained images at two magnification levels with image-level labels. In this work, all Normal and OSCC images are used for binary evaluation. This dataset contains 290 Normal images and 934 OSCC images, with a total of 1224 images. We split the dataset into training, validation, and testing sets with a ratio of 7:1:2. Specifically, Normal is split into Train 203, Val 29, and Test 58, while OSCC is split into Train 654, Val 93, and Test 187.

Dataset 2 is derived from the ORCHID high-resolution oral pathology image resource released by Chaudhary et al. Chaudhary et al. (34) The original collection covers Normal and multiple oral lesion categories and provides large-scale patch-level images. We only select the categories related to Normal and OSCC, and merge OSCC samples of different grades into a single OSCC class for binary evaluation, in order to examine the stability of the proposed method under a larger scale and stronger staining variations. Under our setting, this dataset contains 1502 Normal images and 10188 OSCC images, with a total of 11690 images. We use the same 7:1:2 split for training, validation, and testing. Specifically, Normal is split into Train 1051, Val 150, and Test 301, while OSCC is split into Train 7132, Val 1019, and Test 2037. As summarized in **Table 1**, we report the dataset statistics and the train/val/test splits for both Normal and OSCC.

**Table 1:** Statistics and splits of the oral cancer histopathology image datasets used in this work.

Dataset	Class	Total	Train	Val	Test
Rahman dataset	Normal	290	203	29	58
Rahman dataset	OSCC	934	654	93	187
ORCHID dataset	Normal	1502	1051	150	301
ORCHID dataset	OSCC	10188	7132	1019	2037
Dataset		Total	Train	Val	Test
Rahman dataset		1224	857	122	245
ORCHID dataset		11690	8183	1169	2338

To further assess the real-world generalization of the proposed method, we additionally perform an external validation on an independent retrospective cohort collected from a local hospital. This external cohort contains 500 H&E-stained oral histopathology images with image-level labels for binary classification (Normal vs OSCC), including 400 Normal images and 100 OSCC images. Importantly, this cohort is used only for external testing, and no image from the external cohort is involved in model training, validation, hyper-parameter tuning, or any form of model selection. All data are de-identified prior to analysis, and the study is conducted in compliance with applicable local laws and institutional regulations governing retrospective research using anonymized medical data. The study protocol has been reviewed and approved by the Research Ethics Committee of Panzhihua Central Hospital (Approval No. 2025-069). Given the retrospective nature of the study and the use of anonymized data, the requirement for informed consent was waived by the ethics committee. We report the external validation results using the same evaluation metrics as the internal testing to ensure a fair and consistent comparison under different acquisition conditions.

### 3.2 Overall model architecture

**Figure 1:**
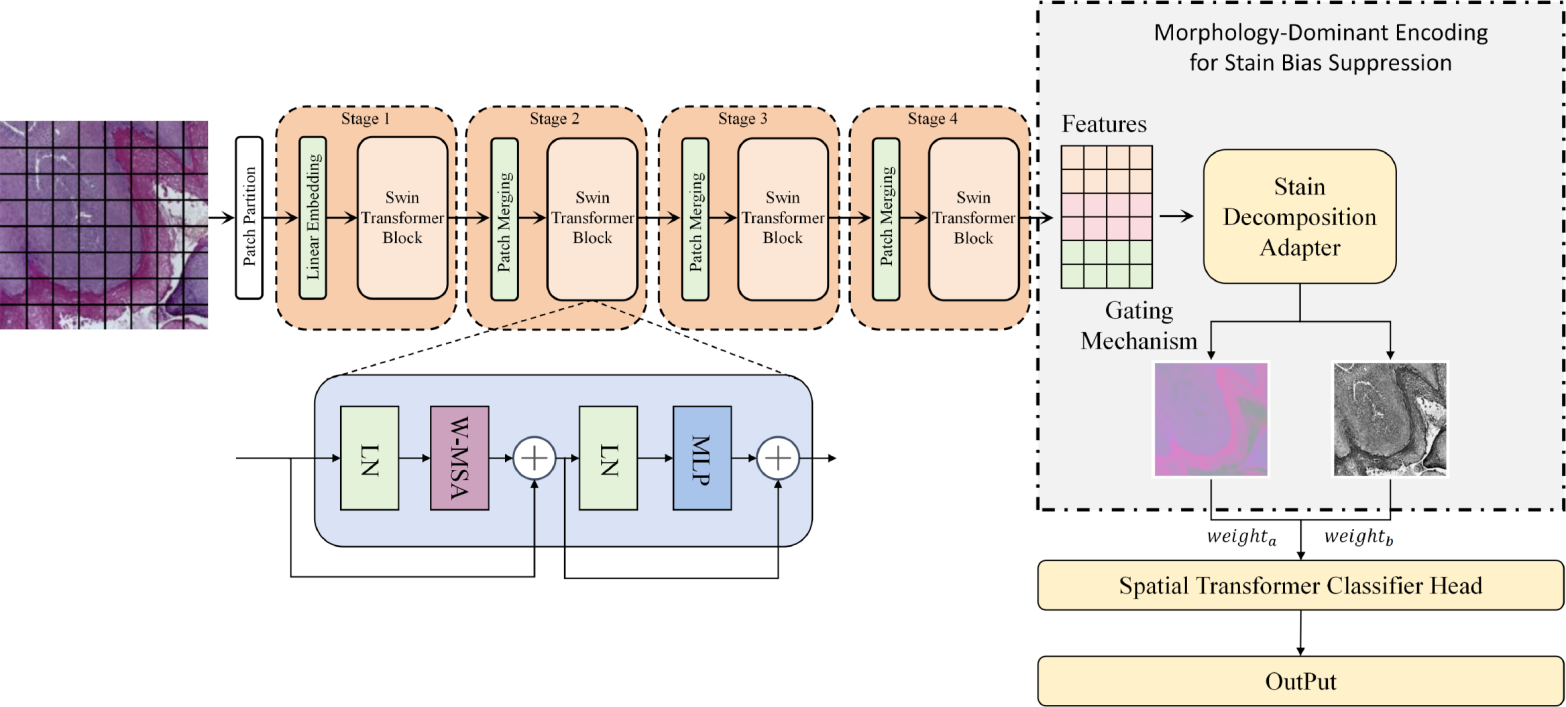
The overall framework takes a histopathology image as input and uses a Swin Transformer backbone to extract token-level feature representations. A morphology dominant encoding module with staining-bias suppression is then applied after the backbone to produce morphology-enhanced robust features via stain decomposition and adaptive gating, which are finally fed into a spatial Transformer classification head for Normal vs. OSCC prediction.

The proposed model consists of three components, including a feature backbone, a morphology-dominant encoding module with staining-bias suppression, and a spatial classification head, aiming at binary classification of oral squamous cell carcinoma (OSCC) histopathology images. The overall architecture is illustrated in **Figure 1**. Given an input image $x\in {\mathbb{R}}^{H\times W\times 3}$, we first obtain a token sequence via patch partition and linear embedding, and then feed it into a hierarchical Swin Transformer backbone to learn multi-scale representations. The backbone outputs a token-level feature matrix at the last stage as shown in Equation 1:

(1) $$F={\text{Φ}}_{\text{Swin}}(x),\;F\in {\mathbb{R}}^{N\times C},$$

where *N* denotes the number of tokens corresponding to spatial grid locations, *C* denotes the channel dimension, and Φ_Swin_(·) is the backbone feature extraction mapping. Different from conventional practices that place staining processing at the input side, we perform staining-bias suppression after the backbone features. This design makes the model less sensitive to color shifts caused by inter-laboratory staining differences while preserving structural information, and is therefore more consistent with the cross-source distribution characteristics of real clinical data.

After obtaining F, we introduce the morphology-dominant encoding module with staining-bias suppression to explicitly decompose and adaptively reweight the features. This module contains a stain-decomposition adapter and a gating mechanism, which generate a stain-related component $F_{s}$ and a morphology-dominant component $F_{m}$, and predict gating weights α to highlight morphological cues while suppressing staining perturbations. The resulting robust features for classification are as shown in Equation 2:

(2) $$F_{s}={\text{Ψ}}_{s}(F),\;F_{m}={\text{Ψ}}_{m}(F),\;\tilde{F}=\alpha ⊙F_{m}+(1-\alpha )⊙F_{s},$$

where ${\text{Ψ}}_{s}(\cdot )$ and ${\text{Ψ}}_{m}(\cdot )$ are two learnable feature transformations, $\alpha \in {\left[0,1\right]}^{N\times 1}$ is the token-level gating weight, and ⊙ denotes element-wise multiplication. We then feed $\tilde{F}$ into a spatial Transformer classification head to model contextual dependencies among tokens, and output class probabilities through global aggregation and a linear classifier. Since the classification head operates on morphology-dominant representations with staining effects suppressed, the model can prioritize nuclear morphology, boundary continuity, and tissue structural patterns even under substantial color variations, thereby improving classification stability and interpretability.

### 3.3 Morphology-dominant encoding for stain bias suppression

**Figure 2:**
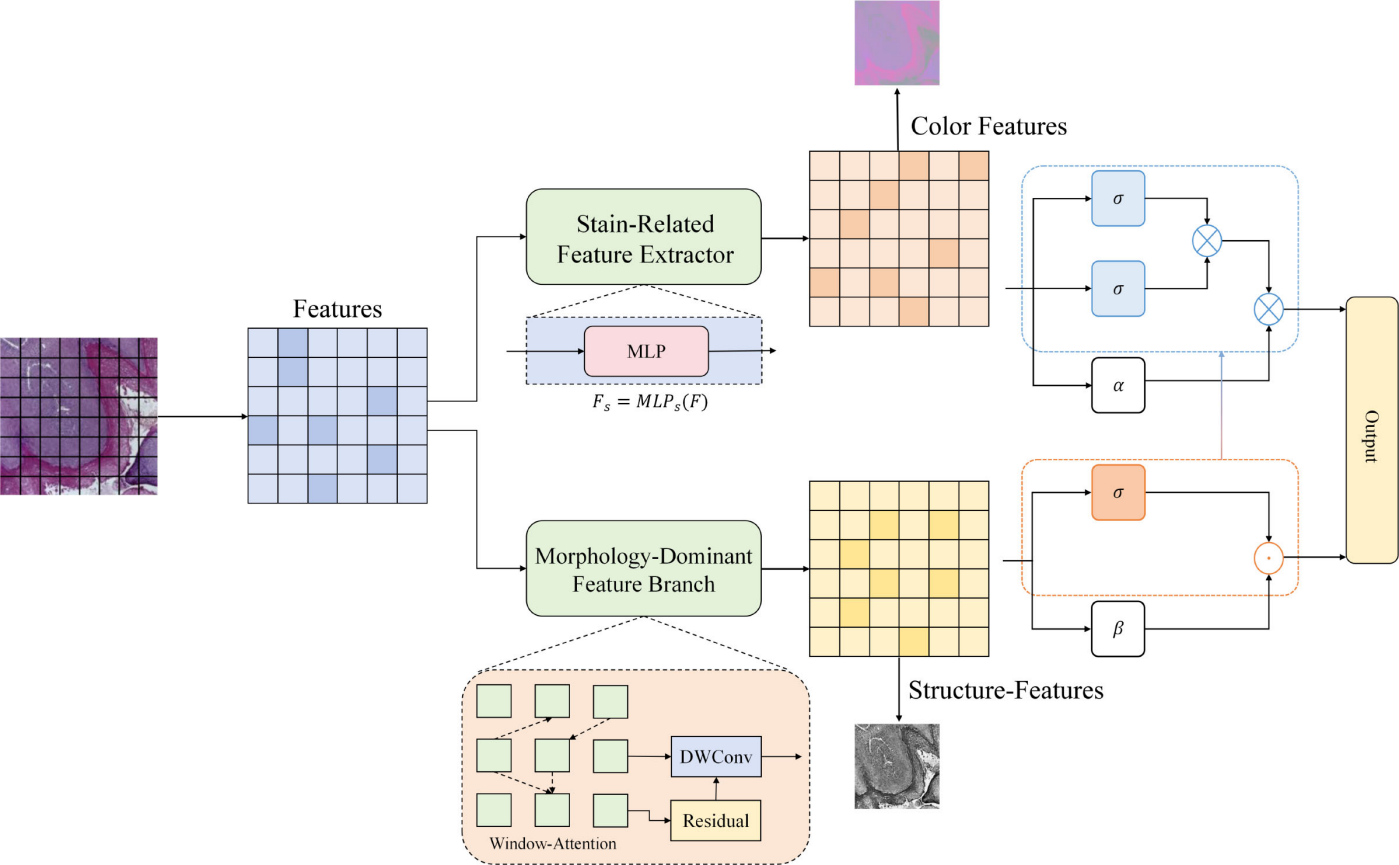
The feature-space processing pipeline of the morphology-dominant encoding module with staining-bias suppression is as follows. The token features output by the backbone are decomposed into a stain-related component and a morphology-dominant component, which form response maps for color features and structural features, respectively. An adaptive gating weight is then used to reweight and fuse the two components, producing morphology-structure dominant robust features that are fed to the downstream classifier.

Histopathology images of oral squamous cell carcinoma (OSCC) often exhibit pronounced chromatic drift and brightness discrepancies across different laboratory staining protocols and scanning devices. Such variations may drive a model to over-rely on color cues while ignoring more medically meaningful nuclear morphology and tissue architectural patterns; the module architecture is illustrated in **Figure 2**.

To improve robustness under diverse staining conditions, we introduce a morphology-dominant encoding module after the token features output by the backbone network, shifting representation learning from input-domain color alignment to feature-domain factorized modeling. Let the backbone output token-level features be $F\in {\mathbb{R}}^{N\times C}$, where *N* is the number of tokens and corresponds one-to-one with spatial grid locations, and *C* is the channel dimension. We first explicitly decompose **F** into a stain-related component and a structure-related component, which provides a controllable representational basis for subsequent adaptive suppression as shown in Equations 3, 4, 5:

(3) $$F=\text{Φ}(x),\;F\in {\mathbb{R}}^{N\times C},$$

(4) $$F_{s}={\text{Ψ}}_{s}(F),\;F_{s}\in {\mathbb{R}}^{N\times C},$$

(5) $$F_{m}={\text{Ψ}}_{m}(F),\;F_{m}\in {\mathbb{R}}^{N\times C},$$

where ${\text{Ψ}}_{s}(\cdot )$ is the stain-related feature extraction mapping that captures response variations induced by chromatic perturbations and background staining differences, and ${\text{Ψ}}_{m}(\cdot )$ is the morphology-dominant feature branch that emphasizes stable cues such as nuclear boundary morphology, textural details, and local tissue structures. Since this decomposition is conducted in the feature space, it does not rely on external stain templates or explicit color matching, thereby avoiding potential artifacts introduced by preprocessing and preserving discriminative information that is more favorable for pathological structure recognition.

The stain-related component $F_{s}$ is implemented via a lightweight token-wise mapping to control its expressive capacity and to avoid implicitly encoding structural cues in this component. Specifically, we realize it as a per-token feed-forward transformation, which can be written as shown in Equation 6.

(6) $$F_{s}={\text{MLP}}_{s}(F),$$

where MLP*_s_*(·) operates independently on each token and mainly models changes in color distribution and staining intensity. In contrast, the morphology-dominant branch Ψ*_m_*(·) performs explicit local-structure modeling on the token grid, enhancing structural consistency and boundary information within spatial neighborhoods through window attention and depthwise separable convolution. To avoid disrupting the backbone representations and to stabilize training, the morphology branch adopts a residual form to purify and strengthen the features. Its overall mapping can be summarized as shown in Equation 7.

(7) $$F_{m}=F+\text{DWConv}(\text{WAttn}(F)),$$

where WAttn(·) denotes attention interactions within local windows to capture short-range dependencies of tissue structures, and DWConv(·) denotes depthwise separable convolution to inject local texture and boundary sensitivity. This combination encourages the morphology dominant branch to learn structural features that are less sensitive to staining variations.

After obtaining $F_{s}$ and $F_{m},$ we introduce an adaptive gating mechanism to reweight and fuse the two components, achieving staining-bias suppression and morphology prioritization. The gating weights are predicted at the token level, allowing the model to dynamically adjust the contributions of color and structural information according to the strength of evidence at different spatial locations. We define the gating weights as shown in Equation 8.

(8) $$\alpha =\sigma (Fw_{\alpha }+b_{\alpha }),\;\alpha \in {\left[0,1\right]}^{N\times 1},$$

where σ(·) is the Sigmoid function, and $w_{\alpha }\in {\mathbb{R}}^{C\times 1}$ and $b_{\alpha }\in {\mathbb{R}}^{1}$ are learnable parameters. Finally, the robust features produced by the morphology-dominant encoding are obtained via gated fusion as shown in Equation 9:

(9) $$\tilde{F}=\alpha ⊙F_{m}+(1-\alpha )⊙F_{s},$$

where ⊙ denotes element-wise multiplication with broadcasting along the channel dimension. This fusion treats the morphology component as the primary information source and introduces the stain-related component only when necessary to complement local color contrast and background discrimination, thereby maintaining a more stable decision basis under inter-laboratory staining variations and imaging-condition changes. Subsequently, $\tilde{F}$ is fed into the spatial Transformer classification head, enabling the classifier to further model contextual dependencies in a unified token representation space and to output the final Normal versus OSCC predictions.

### 3.4 Spatial transformer classifier head

**Figure 3:**
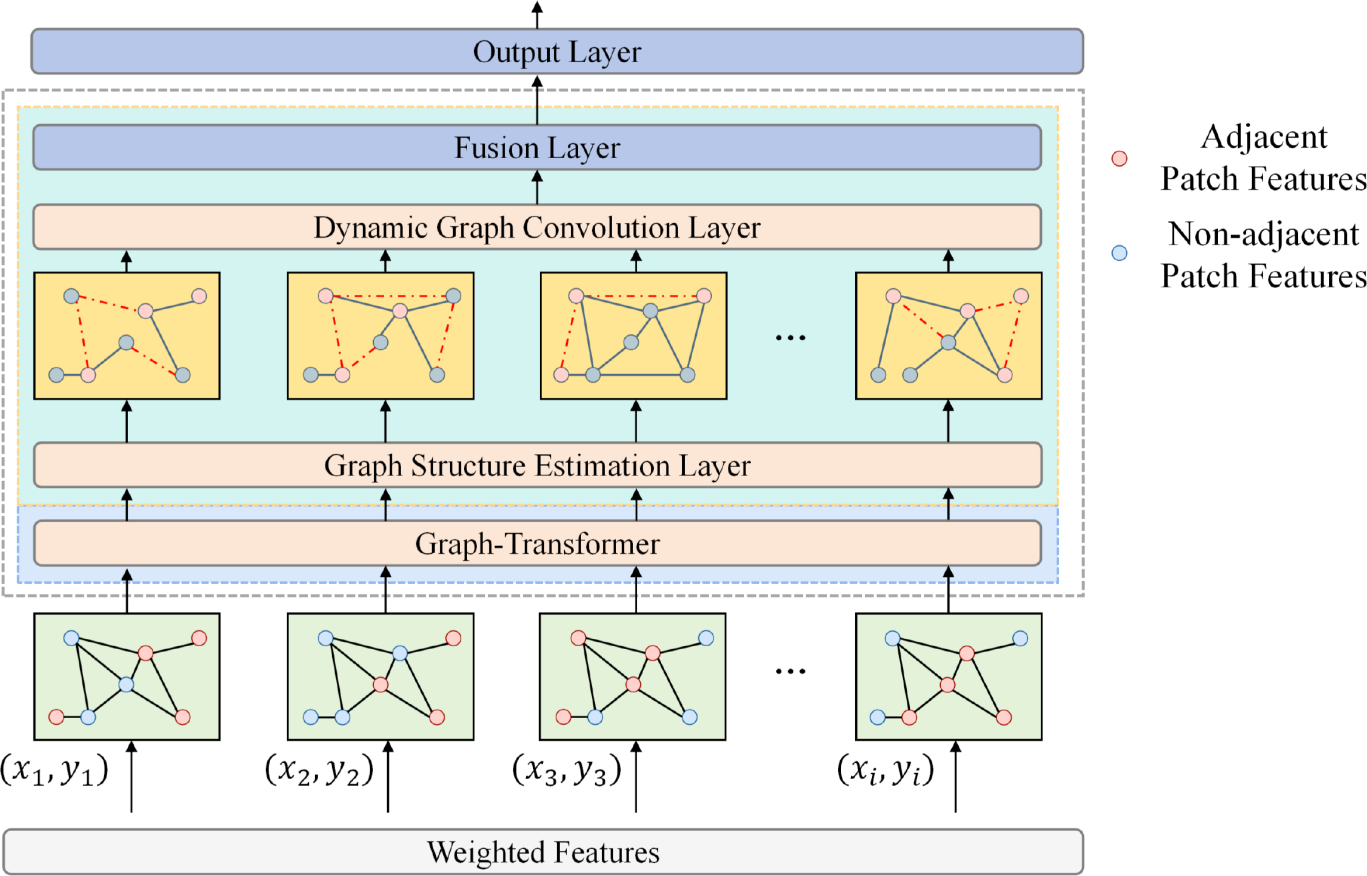
The structured aggregation pipeline of the spatial transformer classification head treats coordinate-aware patch features as graph nodes, jointly models adjacent and non-adjacent relations via graph structure estimation and dynamic graph convolution, and completes global prediction through fusion and the output layer.

After obtaining the robust features produced by the morphology-dominant encoding module, $\tilde{F}\in {\mathbb{R}}^{N\times C}$, we design a spatial Transformer classification head to further model contextual dependencies and spatial topological relations among patch-level instances, so that both local structural evidence and global tissue patterns can be exploited for image-level prediction. Unlike aggregation schemes that perform unstructured weighted summation over instances, this head treats each token as a graph node and explicitly introduces 2D coordinates as a geometric prior, enabling the model to distinguish continuous structures in neighboring regions from semantic complementarity across distant regions; the module architecture is shown in **Figure 3**.

Let the spatial coordinate of the *i*-th instance be $p_{i}=(x_{i},y_{i})$. The geometric embedding is mapped as shown in Equation 10.

(10) $$e_{i}=\text{PE}(p_{i})W_{p}\in {\mathbb{R}}^{d},$$

where PE(·) denotes a learnable or sinusoidal positional encoding, and $W_{p}$ is a linear mapping matrix. By fusing the geometric embedding with the token features, we obtain a spatially aware node representation as shown in Equation 11.

(11) $$h_{i}^{\left(0\right)}={\tilde{f}}_{i}W_{f}+e_{i},\;i=1,\ldots ,N,$$

where ${\tilde{f}}_{i}$ is the *i*-th row of $\tilde{F}$ and $W_{f}$ is the feature projection matrix. To reflect both local continuity and long-range interactions in attention, we decompose the adjacency relations into adjacent edges and non-adjacent edges, and model them separately in subsequent structure estimation and message passing.

To construct spatial topology constraints, we first form a candidate adjacency set based on geometric distances. Let the distance between two nodes be $d_{ij}=\|p_{i}-p_{j}{\|}_{2}$ and define the adjacency indicator matrices as shown in Equation 12.

(12) $$A_{ij}^{\text{adj}}=I(d_{ij}\leq r),\;A_{ij}^{\text{non}}=1-A_{ij}^{\text{adj}},$$

where *r* is the neighborhood radius and I(·) is the indicator function. Since geometric adjacency alone is insufficient to capture long-range associations among lesions of the same type, we further introduce content-based dynamic structure estimation to adaptively refine edge strengths. Specifically, we compute similarity scores for node pairs by as shown in Equation 13.

(13) $$S_{ij}=\frac{(h_{i}^{\left(0\right)}W_{q}){\left(h_{j}^{\left(0\right)}W_{k}\right)}^{⊤}}{\sqrt{d}},$$

where $W_{q},W_{k}$ are projection matrices and *d* is the projection dimension. We then normalize the scores within the adjacent and non-adjacent sets, respectively, to obtain two types of dynamic adjacency weights as shown in Equation 14.

(14) $${\hat{A}}_{ij}^{\text{adj}}=\frac{\text{exp}(S_{ij})A_{ij}^{\text{adj}}}{\sum_{k}\text{exp}(S_{ik})A_{ik}^{\text{adj}}},\;{\hat{A}}_{ij}^{\text{non}}=\frac{\text{exp}(S_{ij})A_{ij}^{\text{non}}}{\sum_{k}\text{exp}(S_{ik})A_{ik}^{\text{non}}},$$

and fuse them via gating to obtain the final structure estimation matrix as shown in Equation 15.

(15) $${\hat{A}}_{ij}=\lambda \;{\hat{A}}_{ij}^{\text{adj}}+(1-\lambda )\;{\hat{A}}_{ij}^{\text{non}},$$

where *λ* ∈ [0,1] is a learnable coefficient that adaptively balances local continuous structures and long-range semantic complementarity. This structure estimation enables the classification head to emphasize morphological continuity in adjacent regions while capturing associations between the same tumor nest or similar tissue morphology at distant locations.

After obtaining $\hat{A},$ we adopt a cooperative update mechanism that combines a graph Transformer and dynamic graph convolution, and iteratively update node representations across multiple layers to achieve structured aggregation. The graph Transformer attention update at layer *ℓ* as shown in Equation 16.

(16) $$h_{i}^{\left(l+\frac{1}{2}\right)}=\sum_{j=1}^{N}{\hat{A}}_{ij}\;{\text{softmax}}_{j}(\frac{(h_{i}^{\left(l\right)}W_{q}^{l}){\left(h_{j}^{\left(l\right)}W_{k}^{l}\right)}^{⊤}}{\sqrt{d}})\;(h_{j}^{\left(l\right)}W_{v}^{l}),$$

where $W_{q}^{l},W_{k}^{l},W_{v}^{l}$ are the projection matrices at layer *ℓ*. We then apply dynamic graph convolution to further strengthen structural consistency within neighborhoods and suppress noise. The update can be written as as shown in Equation 17.

(17) $$h_{i}^{\left(l+1\right)}=\sigma (\sum_{j=1}^{N}{\hat{A}}_{ij}\;h_{j}^{\left(l+\frac{1}{2}\right)}W_{g}^{l})+h_{i}^{\left(l\right)},$$

where $W_{g}^{l}$ is the graph convolution weight, σ(·)  is a nonlinear function, and the residual term stabilizes deep propagation while preserving the original discriminative information. Finally, we aggregate all node representations into an image-level representation vector and output the binary classification probabilities. We use an attention-based global readout as the input to the output layer as shown in Equation 18:

(18) $$a_{i}=\frac{\text{exp}(u^{⊤}\text{ tanh}(h_{i}^{\left(L\right)}W_{a}))}{\sum_{k}\text{exp}(u^{⊤}\;\text{tanh}(h_{k}^{\left(L\right)}W_{a}))},\;z=\sum_{i=1}^{N}a_{i}h_{i}^{\left(L\right)},$$

where *L* is the number of layers, $W_{a}$ and **u** are learnable parameters, and **z** is the image-level representation. The classification output is as shown in Equation 19.

(19) $$\hat{y}=\text{softmax}(zW_{c}+b_{c}),$$

where $W_{c}$ and $b_{c}$ are classifier parameters. Since this classification head explicitly injects spatial topology during structure estimation and jointly models local continuity and long-range associations during updates, it can more stably aggregate key lesion evidence from morphology-dominant features and provide consistent patch-level response cues for subsequent interpretability analysis.

## 4 Evaluation metrics

To comprehensively evaluate the performance of the proposed model on the binary classification task between Normal and OSCC, we adopt Accuracy (Acc), Precision, Recall, F1-Score, and the area under the curve (AUC) as evaluation metrics. The definition, corresponding formula, and the meaning of symbols for each metric are given as follows.

### 4.1 Accuracy

Accuracy measures the overall proportion of correct predictions, defined as the ratio of correctly predicted samples to the total number of samples as shown in Equation 20:

(20) $$\text{Acc}=\frac{TP+TN}{TP+TN+FP+FN}.$$

Here, *TP* (True Positive) denotes the number of positive samples (OSCC) correctly predicted as positive; *TN* (True Negative) denotes the number of negative samples (Normal) correctly predicted as negative; *FP* (False Positive) denotes the number of negative samples incorrectly predicted as positive; and *FN* (False Negative) denotes the number of positive samples incorrectly predicted as negative.

### 4.2 Precision

Precision measures the proportion of true positives among all samples predicted as positive, reflecting the reliability of positive predictions as shown in Equation 21:

(21) $$\text{Precision}=\frac{TP}{TP+FP}.$$

Here, *TP* denotes the number of true positives, i.e., samples that are truly OSCC and are predicted as OSCC; *FP* denotes the number of false positives, i.e., samples that are truly Normal but are predicted as OSCC.

### 4.3 Recall

Recall measures the proportion of true positives among all actual positive samples, reflecting the coverage of positive samples as shown in Equation 22:

(22) $$\text{Recall}=\frac{TP}{TP+FN}.$$

Here, *TP* denotes the number of true positives, i.e., samples that are truly OSCC and are predicted as OSCC; *FN* denotes the number of false negatives, i.e., samples that are truly OSCC but are predicted as Normal.

### 4.4 F1-Score

F1-Score is the harmonic mean of Precision and Recall, which balances Precision and Recall and is suitable for scenarios with class imbalance or when both missed detections and false alarms are of concern as shown in Equation 23:

(23) $$\text{F}1=\frac{2\times \text{Precision}\times \text{Recall}}{\text{Precision}+\text{Recall}}.$$

Here, Precision denotes precision and Recall denotes recall, which characterize the correctness and coverage of positive predictions, respectively.

### 4.5 AUC

AUC (Area Under the ROC Curve) denotes the area under the ROC curve and measures the overall ability of the model to separate positive and negative classes under different decision thresholds. It can be defined as the integral area of the ROC curve as shown in Equation 24:

(24) $$\text{AUC}=\int_{0}^{1}\text{TPR}(u)\text{dFPR}(u).$$

Here, TPR(u) denotes the true positive rate (TPR) at threshold *u*, and FPR(u) denotes the false positive rate (FPR) at threshold *u*. They are defined as: $\text{TPR}=\frac{TP}{TP+FN}$ and $\text{FPR}=\frac{FP}{FP+TN}$. A larger AUC indicates stronger separability between OSCC and Normal.

## 5 Experimental results and analysis

### 5.1 Experimental setup

We conduct binary classification experiments between Normal and OSCC on two public oral cancer histopathology image datasets to validate the effectiveness and robustness of the proposed method under different dataset scales and staining variations. All images are uniformly resized to a resolution of 224 × 224 before being fed into the backbone network, and normalization is applied to stabilize the training process. To alleviate bias introduced by class imbalance, we adopt a stratified sampling strategy for the two classes during training and introduce class weights into the cross-entropy loss during optimization. Model parameters are updated using the AdamW optimizer, and the learning rate is gradually decayed with a cosine annealing schedule. During training, the best checkpoint is selected according to the validation set performance and is used for the final test evaluation. To ensure reproducibility, all experiments fix the random seed and are repeated under the same training configuration.

In terms of implementation details, the backbone network is a hierarchical Swin Transformer with patch size set to 4 and window size set to 7. ImageNet-pretrained weights are used for initialization to accelerate convergence and improve generalization. The morphology-dominant encoding module with staining-bias suppression and the spatial Transformer classification head are deployed sequentially after the backbone output features, and are jointly optimized with the backbone in an end-to-end manner. Mixed-precision training is adopted to improve memory utilization and training efficiency, and performance metrics on the validation set are computed after each epoch to monitor convergence and potential overfitting. **Table 2** summarizes the main software/hardware environment and key hyperparameter settings used in this work.

**Table 2:** Software/hardware environment and key hyperparameter settings.

Item	Setting
Operating system	Ubuntu 20.04
CPU	Intel Xeon
GPU	NVIDIA RTX 4090 24GB
Memory	64GB
Deep learning framework	PyTorch 2.2
CUDA	CUDA 12.1
Input resolution	224 × 224
Batch size	32
Epochs	200
Optimizer	AdamW
Initial learning rate	1 × 10^−4^
Weight decay	1 × 10^−2^
Learning rate schedule	Cosine annealing
Gradient clipping	1.0
Random seed	42
Mixed precision	FP16
Swin patch size	4
Swin window size	7

### 5.2 Experimental results compared with other models

To comprehensively validate the effectiveness of the proposed method for oral squamous cell carcinoma histopathology image binary classification and its practicality for real-world deployment, we construct a broad comparison benchmark. First, we include traditional machine learning classifiers such as XGBoost Chen (35) and Decision Tree Loh (36) as lightweight baselines to characterize the achievable performance upper bound under weak representation settings. Second, we introduce generic deep visual backbones, including ResNet50 He et al. (37), VGG19 Simonyan and Zisserman (38), ResNeXt Xie et al. (39), ConvNeXt Liu et al. (40), ConvNeXtV2 Woo et al. (41), and ViT Dosovitskiy (42), to evaluate the performance of common end-to-end feature learning frameworks on this task. Furthermore, we incorporate representative methods designed for medical imaging or structural modeling, including EcgMLP Sheakh et al. (43), DraCN Tursun et al. (44), MedTrans Xu et al. (45), CLASH Rather and Kumar (46), EFFResNet-ViT Hussain et al. (47), IncARMAG Remigio (48), Mscpt Han et al. (49), and DVPT He et al. (50), to examine how specialized architectural designs adapt to pathology scenarios. The evaluation metrics cover both classification accuracy and robustness-related measures (Acc, Precision, Recall, F1-Score, AUC) as well as inference efficiency (FPS), thereby establishing a comparable benchmark along both performance and speed dimensions and providing a basis for subsequent analysis of where the proposed method gains advantages across different modeling paradigms. We first report the experimental results on Dataset 1, as shown in **Table 3**.

**Table 3:** Comparison results with traditional machine learning baselines, generic deep models, and medical image classification methods.

Method	Acc	Precision	Recall	F1-score	AUC	FPS
XGBoost	74.82 ± 0.413	88.31 ± 0.427	72.40 ± 0.452	79.41 ± 0.439	82.73 ± 0.418	–
Decision Tree	68.57 ± 0.536	84.92 ± 0.501	66.35 ± 0.548	74.53 ± 0.521	78.64 ± 0.493	–
ResNet50	79.44 ± 0.382	91.07 ± 0.365	77.83 ± 0.409	84.01 ± 0.392	88.92 ± 0.371	65.2
VGG19	76.82 ± 0.471	89.54 ± 0.452	74.66 ± 0.498	81.46 ± 0.463	86.71 ± 0.451	58.4
ResNeXt	81.33 ± 0.393	92.18 ± 0.372	79.12 ± 0.421	85.05 ± 0.404	90.31 ± 0.387	61.7
ConvNeXt	80.46 ± 0.402	91.63 ± 0.384	78.54 ± 0.439	84.51 ± 0.418	89.74 ± 0.396	59.8
ConvNeXtV2	81.77 ± 0.377	92.45 ± 0.361	79.44 ± 0.395	85.23 ± 0.382	90.86 ± 0.368	58.9
ViT	78.93 ± 0.418	90.73 ± 0.402	77.12 ± 0.447	83.17 ± 0.428	88.11 ± 0.407	52.6
EcgMLP	82.64 ± 0.341	93.12 ± 0.335	80.54 ± 0.372	86.28 ± 0.354	91.44 ± 0.329	140.3
DraCN	74.32 ± 0.463	88.06 ± 0.446	72.02 ± 0.489	79.05 ± 0.468	82.34 ± 0.452	48.1
MedTrans	79.17 ± 0.391	90.82 ± 0.374	77.55 ± 0.421	83.59 ± 0.403	88.63 ± 0.382	46.7
CLASH	80.88 ± 0.354	91.91 ± 0.341	78.93 ± 0.376	85.07 ± 0.362	90.02 ± 0.347	47.2
EFFResNet-ViT	83.25 ± 0.318	93.36 ± 0.302	81.27 ± 0.349	86.59 ± 0.331	92.15 ± 0.315	44.3
IncARMAG	84.72 ± 0.292	94.02 ± 0.279	82.44 ± 0.318	88.06 ± 0.301	93.81 ± 0.288	43.6
Mscpt	85.51 ± 0.271	94.57 ± 0.262	83.39 ± 0.301	88.88 ± 0.284	94.26 ± 0.273	42.8
DVPT	84.93 ± 0.286	94.22 ± 0.271	82.77 ± 0.312	88.32 ± 0.297	93.94 ± 0.284	42.1
Ours	87.35 ± 0.351	96.43 ± 0.338	86.63 ± 0.379	91.27 ± 0.361	98.04 ± 0.342	41.8

As shown in **Table 3**, the performance of different paradigms on this task exhibits a clear gradient. Traditional machine learning methods (e.g., XGBoost and Decision Tree) achieve relatively low overall performance, indicating that without strong representation learning capability it is difficult to sufficiently capture the complex morphological and structural differences in histopathology images. Generic deep backbones (ResNet50, VGG19, ResNeXt, ConvNeXt, ConvNeXtV2, and ViT) generally outperform traditional baselines in terms of accuracy- and AUC-related metrics, but noticeable gaps still remain across metrics, suggesting that this task is sensitive to local heterogeneity and staining variations and that relying solely on end-to-end feature learning may still lead to unstable decisions. Specialized models designed for medical imaging or structural modeling (e.g., EcgMLP, CLASH, EFFResNet-ViT, IncARMAG, Mscpt, and DVPT) further improve the overall performance, demonstrating that stronger structural representations and aggregation mechanisms help extract key lesion evidence. Building upon these baselines, our method achieves the best results in Acc, Precision, Recall, F1-Score, and AUC, with a particularly higher AUC, while maintaining an inference speed comparable to other complex models. These results indicate that the proposed morphology-dominant feature suppression strategy and spatial structured modeling can more effectively focus on discriminative regions and reduce interference from non-critical factors, thereby achieving a more favorable balance between performance gains and computational overhead. Further experimental results for dataset 2 are presented in **Table 4**.

**Table 4:** Comparison results on dataset 2 with traditional machine learning baselines, generic deep models, and medical image classification methods.

Method	Acc	Precision	Recall	F1-score	AUC	FPS
XGBoost	71.84 ± 0.472	93.12 ± 0.451	70.03 ± 0.498	79.89 ± 0.467	78.42 ± 0.453	–
Decision Tree	65.27 ± 0.563	89.44 ± 0.531	63.41 ± 0.587	74.15 ± 0.552	71.03 ± 0.538	–
ResNet50	74.33 ± 0.418	95.22 ± 0.392	72.14 ± 0.447	82.03 ± 0.423	83.67 ± 0.401	65.2
VGG19	72.56 ± 0.502	93.88 ± 0.461	70.93 ± 0.534	80.41 ± 0.511	81.52 ± 0.488	58.4
ResNeXt	75.42 ± 0.387	95.74 ± 0.362	73.38 ± 0.425	83.51 ± 0.396	84.48 ± 0.379	61.7
ConvNeXt	74.91 ± 0.409	95.31 ± 0.384	72.94 ± 0.452	83.05 ± 0.428	83.92 ± 0.402	59.8
ConvNeXtV2	76.63 ± 0.361	96.04 ± 0.344	74.41 ± 0.402	84.21 ± 0.374	85.33 ± 0.351	58.9
ViT	73.87 ± 0.455	94.36 ± 0.433	71.24 ± 0.481	81.12 ± 0.459	82.44 ± 0.446	52.6
EcgMLP	77.11 ± 0.342	96.38 ± 0.331	75.33 ± 0.381	84.91 ± 0.352	86.43 ± 0.338	140.3
DraCN	70.94 ± 0.513	92.17 ± 0.474	69.17 ± 0.539	78.86 ± 0.522	77.91 ± 0.501	48.1
MedTrans	74.08 ± 0.427	94.81 ± 0.401	72.58 ± 0.453	82.16 ± 0.434	83.03 ± 0.416	46.7
CLASH	75.64 ± 0.394	95.52 ± 0.372	73.66 ± 0.428	83.71 ± 0.405	84.62 ± 0.381	47.2
EFFResNet-ViT	76.81 ± 0.368	96.03 ± 0.351	74.92 ± 0.411	84.97 ± 0.387	85.94 ± 0.372	44.3
IncARMAG	76.92 ± 0.352	96.44 ± 0.336	75.02 ± 0.398	85.08 ± 0.364	86.22 ± 0.349	43.6
Mscpt	77.08 ± 0.339	96.51 ± 0.327	75.17 ± 0.386	85.26 ± 0.344	86.44 ± 0.331	42.8
DVPT	76.44 ± 0.361	96.22 ± 0.348	74.51 ± 0.403	84.71 ± 0.373	85.93 ± 0.359	42.1
Ours	79.34 ± 0.351	97.38 ± 0.338	78.04 ± 0.379	86.86 ± 0.361	90.74 ± 0.342	41.8

As shown in **Table 4**, on the larger-scale Dataset 2 with more pronounced staining variations, the performance gaps among different baselines become further amplified. Traditional machine learning methods, which rely on shallow statistical features, struggle to characterize the complex morphological structures and spatial heterogeneity of pathological tissues. Generic deep backbones improve representation capacity, but under cross-source staining shifts they are still prone to being distracted by color cues, which limits recall and overall decision stability. Medical imaging specific models strengthen structural modeling to some extent, yet their potential cannot be fully realized when explicit suppression of staining bias is insufficient. In contrast, our method remains leading on key metrics such as Acc, F1, and AUC, demonstrating complementary gains brought by two core designs. First, the morphology-dominant encoding with staining-bias suppression performs feature-space decomposition and adaptive gating on backbone features, encouraging the model to prioritize more stable discriminative cues such as nuclear morphology and tissue architecture, thereby reducing the impact of color drift from the source. Second, the spatial Transformer classification head further models cross-patch spatial context and interactions among key regions on the morphology-enhanced features, enabling more effective aggregation of scattered lesion evidence and improving recall and overall AUC. Meanwhile, our method maintains an acceptable inference speed, indicating that stronger robustness and discriminative capability are achieved without introducing excessive computational overhead.

### 5.3 The ablation experiment results of the algorithm in this paper

To verify the contribution of each key component in the proposed method to the overall performance improvement, and to clarify the operating mechanisms of the two core innovations under different data conditions, we conduct a systematic ablation study. Specifically, while keeping the backbone network and training strategy unchanged, we remove or replace the morphology-dominant encoding module with staining-bias suppression and the spatial Transformer classification head, and further compare settings related to gated fusion and spatial modeling to identify the sources of performance variation. All ablation configurations use the same data splits and evaluation metrics as the main experiments, ensuring fair comparisons and reproducible conclusions. The results are reported in **Table 5**.

**Table 5:** Ablation study results on the two datasets.

Dataset 1					
Method	Acc	Precision	Recall	F1-score	AUC
Swin-Transformer	82.45 ± 0.512	95.00 ± 0.488	81.28 ± 0.531	87.61 ± 0.509	93.20 ± 0.476
+SDA	84.20 ± 0.461	95.62 ± 0.452	83.05 ± 0.498	88.86 ± 0.472	95.10 ± 0.431
+STC	85.90 ± 0.412	96.05 ± 0.401	84.92 ± 0.452	90.14 ± 0.428	96.80 ± 0.392
Ours	87.35 ± 0.351	96.43 ± 0.338	86.63 ± 0.379	91.27 ± 0.361	98.04 ± 0.342

Dataset 2					
Method	Acc	Precision	Recall	F1-score	AUC
Swin-Transformer	72.93 ± 0.583	96.06 ± 0.552	71.87 ± 0.601	82.22 ± 0.577	74.05 ± 0.544
+SDA	75.60 ± 0.512	96.58 ± 0.501	74.20 ± 0.556	84.05 ± 0.521	82.50 ± 0.498
+STC	77.80 ± 0.441	97.05 ± 0.421	76.50 ± 0.482	85.81 ± 0.456	87.20 ± 0.415
Ours	79.34 ± 0.351	97.38 ± 0.338	78.04 ± 0.379	86.86 ± 0.361	90.74 ± 0.342

As indicated by the ablation results in **Table 5**, the two core components provide clear and additive contributions to performance improvements, and their gains exhibit different emphases as the staining variations and dataset scale change. On Dataset 1, the Swin-Transformer baseline already has strong representation capability, yet adding SDA still yields consistent improvements across all metrics, indicating that decomposing stain-related perturbations in the feature space and using gating to prioritize morphological cues can further reduce the interference of color bias on classification. After introducing STC, the performance continues to increase, suggesting that a spatial-context-aware Transformer classification head can more effectively aggregate lesion evidence scattered across different patches and improve overall decision consistency. When both components are enabled, the performance reaches the highest level, demonstrating that morphology-dominant robust features provide cleaner and more structured inputs for STC, allowing spatial modeling to focus more on tissue structures rather than staining differences. More importantly, on Dataset 2, which has stronger staining shifts and a larger sample scale, the improvements brought by SDA are more pronounced, especially in terms of AUC and F1, validating the necessity of staining-bias suppression in cross-source scenarios. Building on this, STC further strengthens the modeling of relations among key regions, enabling the final model to improve recall and overall separability while maintaining high precision, which highlights that the two innovations collaboratively enhance model stability from the perspectives of robust representation learning and spatial structured aggregation.

### 5.4 Visualize experimental results

#### 5.4.1 Experimental results of loss function varying with epoch

**Figure 4:**
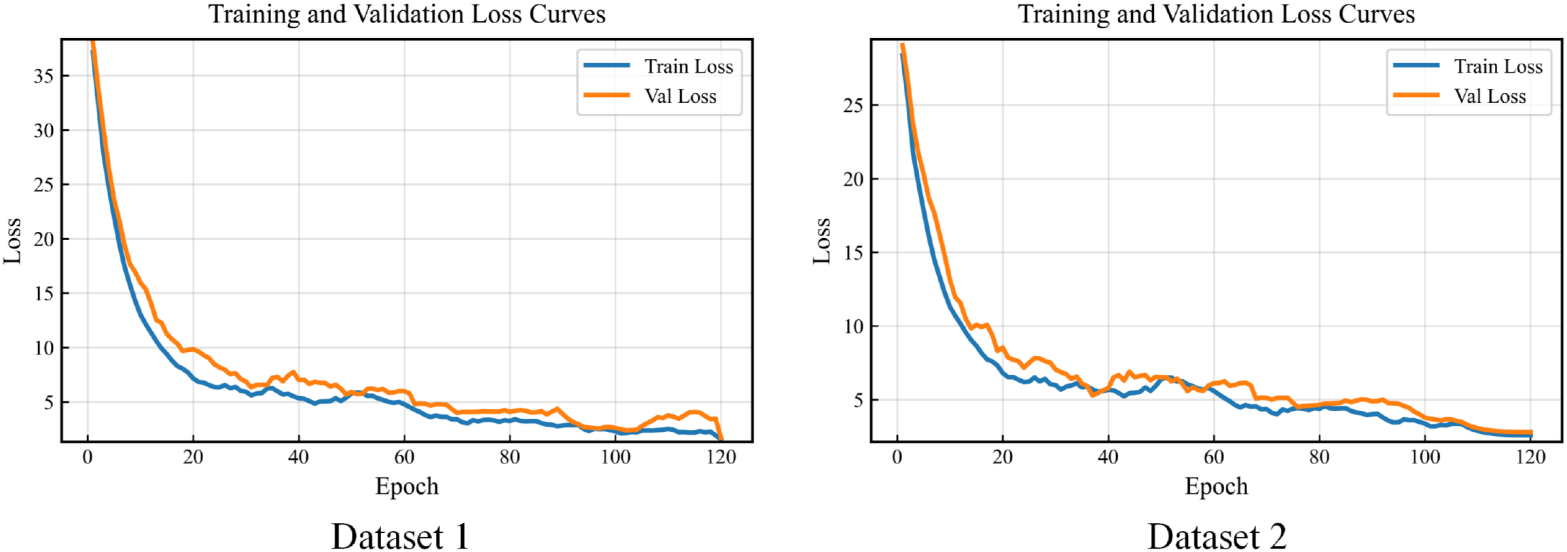
Experimental results of loss function varying with epoch.

To provide an intuitive characterization of the optimization dynamics and convergence behavior during training, we visualize the evolution of the training loss across epochs. By presenting the loss curves under different configurations, we can examine differences in optimization stability from both the overall decreasing trend and the patterns of local fluctuations, and thereby identify potential issues such as oscillations, overfitting, or insufficient convergence. This visualization also offers process-level evidence to support the subsequent discussion of the mechanisms of key modules. The results are shown in **Figure 4**.

From the loss curves, it can be observed that on both datasets the training loss and validation loss drop rapidly in the early stage and gradually become stable in the later stage. Moreover, their overall trends remain closely aligned, without a gap that keeps widening as the number of epochs increases, indicating that the model effectively reduces training error while maintaining relatively stable generalization behavior. In light of the two proposed innovations, this convergence pattern has a clear mechanistic implication: the morphology-dominant encoding with staining-bias suppression weakens the reliance on color-correlated noise in the backbone feature space, so optimization is more focused on structural cues, thereby reducing validation-stage loss fluctuations caused by staining differences. Meanwhile, the spatial Transformer classification head performs structured aggregation of cross-patch spatial context, making gradient updates more consistent and suppressing random oscillations induced by local heterogeneity, which leads to more continuous curves and more controllable convergence in the mid-to-late training stages. Particularly on Dataset 2, which is larger in scale and exhibits stronger staining variations, the validation curve still shows a stable downward trend and converges in sync with the training curve, further demonstrating that the proposed morphology-robust representation and spatial modeling improve optimization stability and convergence reliability under cross-domain staining shifts.

#### 5.4.2 Confusion matrix experimental results

**Figure 5:**
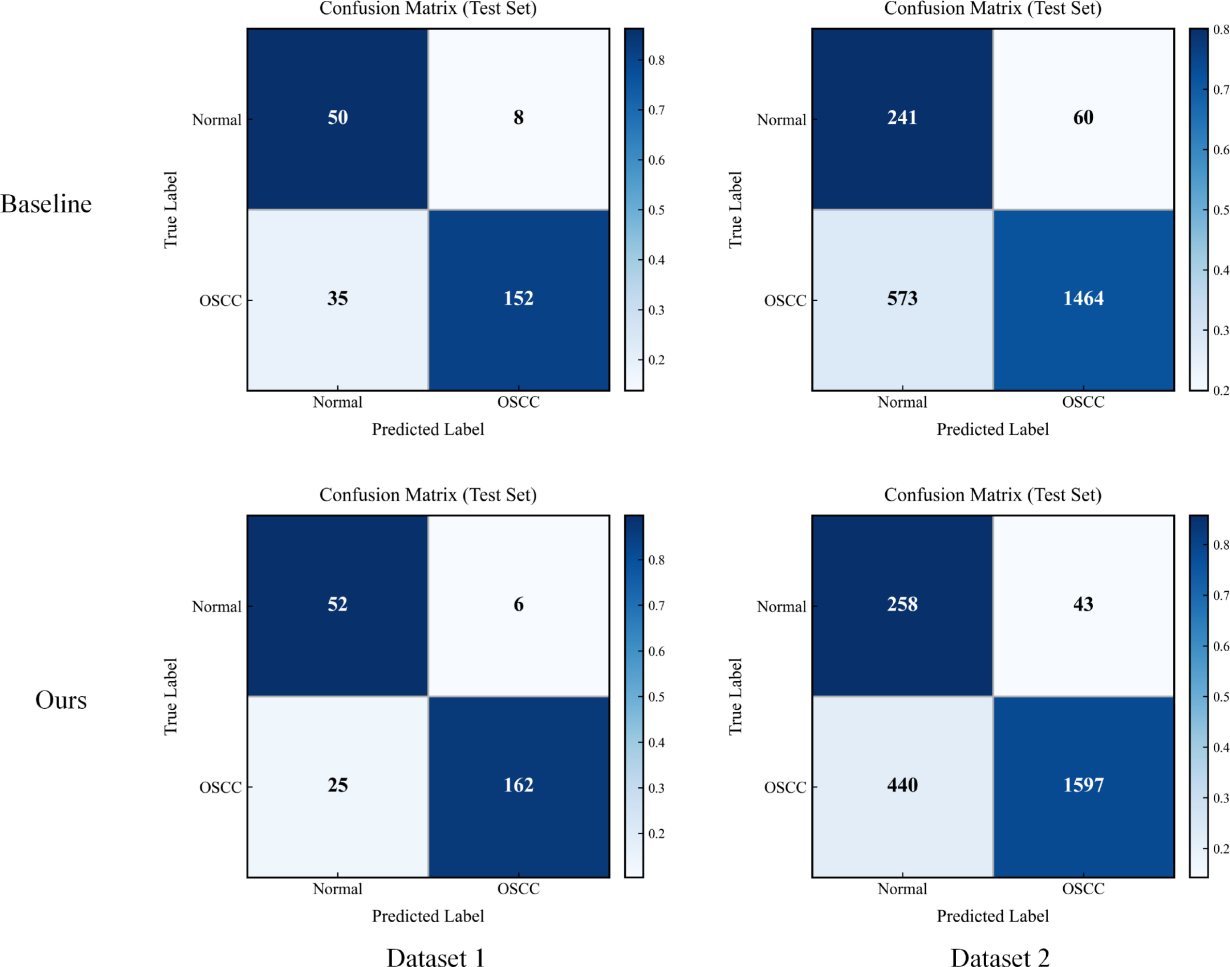
Confusion matrix experimental results.

To characterize the model’s decision behavior on the two classes at a finer granularity, we further visualize the classification outputs using a confusion matrix. Compared with reporting only overall metrics, the confusion matrix provides an intuitive view of the distributions of correct predictions and misclassifications across different classes, thereby revealing the model’s tendencies and potential risk points on positive and negative samples. This result also supports the subsequent discussion on how morphology-dominant encoding and spatial structured aggregation reduce specific types of errors. The results are shown in **Figure 5**.

The confusion matrices more clearly show that the improvement of our method over the baseline is mainly reflected in the simultaneous reduction of both error types, with a more pronounced effect on the clinically critical false negatives of OSCC. On Dataset 1, the number of OSCC samples misclassified as Normal decreases from 35 to 25, while the number of Normal samples misclassified as OSCC decreases from 8 to 6. On the larger-scale Dataset 2 with stronger staining variations, OSCC false negatives are substantially reduced from 573 to 440, and Normal false positives also decrease from 60 to 43. From the perspective of the proposed innovations, the morphology-dominant encoding with staining-bias suppression weakens color-driven spurious correlations in the backbone feature space and encourages the model to rely more on stable cues such as nuclear morphology and tissue architecture, thereby reducing cross-class confusion caused by staining drift. Meanwhile, the spatial Transformer classification head further leverages cross-patch spatial context and structured interactions to strengthen the aggregation of discriminative evidence, making borderline samples less likely to be pushed toward the wrong class by local noise. Overall, this distributional change, which reduces both false positives and false negatives, indicates that the two designs provide complementary synergy, improving sensitivity to OSCC while enhancing specificity to Normal, and thus making the model risk more controllable in practical screening scenarios.

#### 5.4.3 t-SNE experimental results

**Figure 6:**
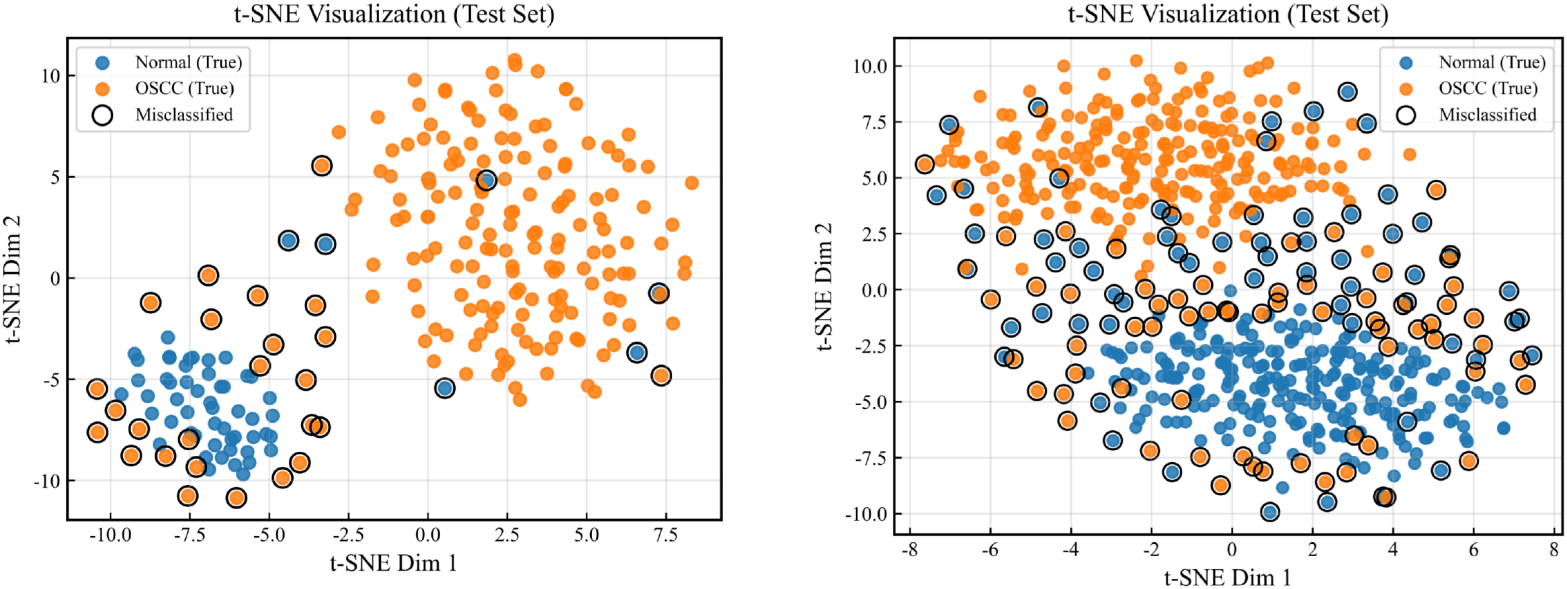
t-SNE experimental results.

To further understand the model’s discriminative mechanism from the perspective of the feature space, we employ t-SNE to perform dimensionality reduction and visualization of the high-dimensional features of test samples. This visualization provides an intuitive view of the clustering structure of different classes and the shape of inter-class boundaries in the embedding space, thereby reflecting feature separability and potential overlap regions. By comparing the distribution differences across different models or different module configurations, it offers direct evidence for the representation improvements brought by morphology-dominant encoding and spatial structured aggregation. The results are shown in **Figure 6**.

The t-SNE visualizations provide an intuitive view of how the model organizes classes in the feature space. On Dataset 1, the two classes form more compact clusters with a clearer separation, and most misclassified points appear in the transitional region between the two clusters, indicating that the learned decision boundary is relatively well defined and that the main difficulty comes from a small number of borderline samples with similar morphology. In contrast, on Dataset 2, due to its larger scale and stronger staining shifts, the feature distribution exhibits more obvious inter-class overlap and long-tail scattered points, and misclassified samples are more likely to fall into the overlap band, suggesting that this dataset imposes higher requirements on representational robustness and spatial consistency. In terms of the proposed innovations, the morphology-dominant encoding with staining-bias suppression can decouple features from color-dominated variations, allowing samples from the same class to converge more easily toward a structurally consistent direction in the embedding space and thus reducing intra-class dispersion caused by staining differences. Meanwhile, the spatial Transformer classification head strengthens the associations among local evidence through structured cross-patch aggregation, making multiple patches related to the same lesion more consistent at the feature level, which further improves cluster compactness and compresses the inter-class overlap region. Overall, this visualization supports the mechanistic hypothesis of our method from a representation learning perspective, namely that morphology-dominant robust representations and spatial context modeling jointly enhance class separability and confine hard samples more tightly to boundary regions rather than causing widespread confusion.

#### 5.4.4 Grad-cam experimental results

**Figure 7:**
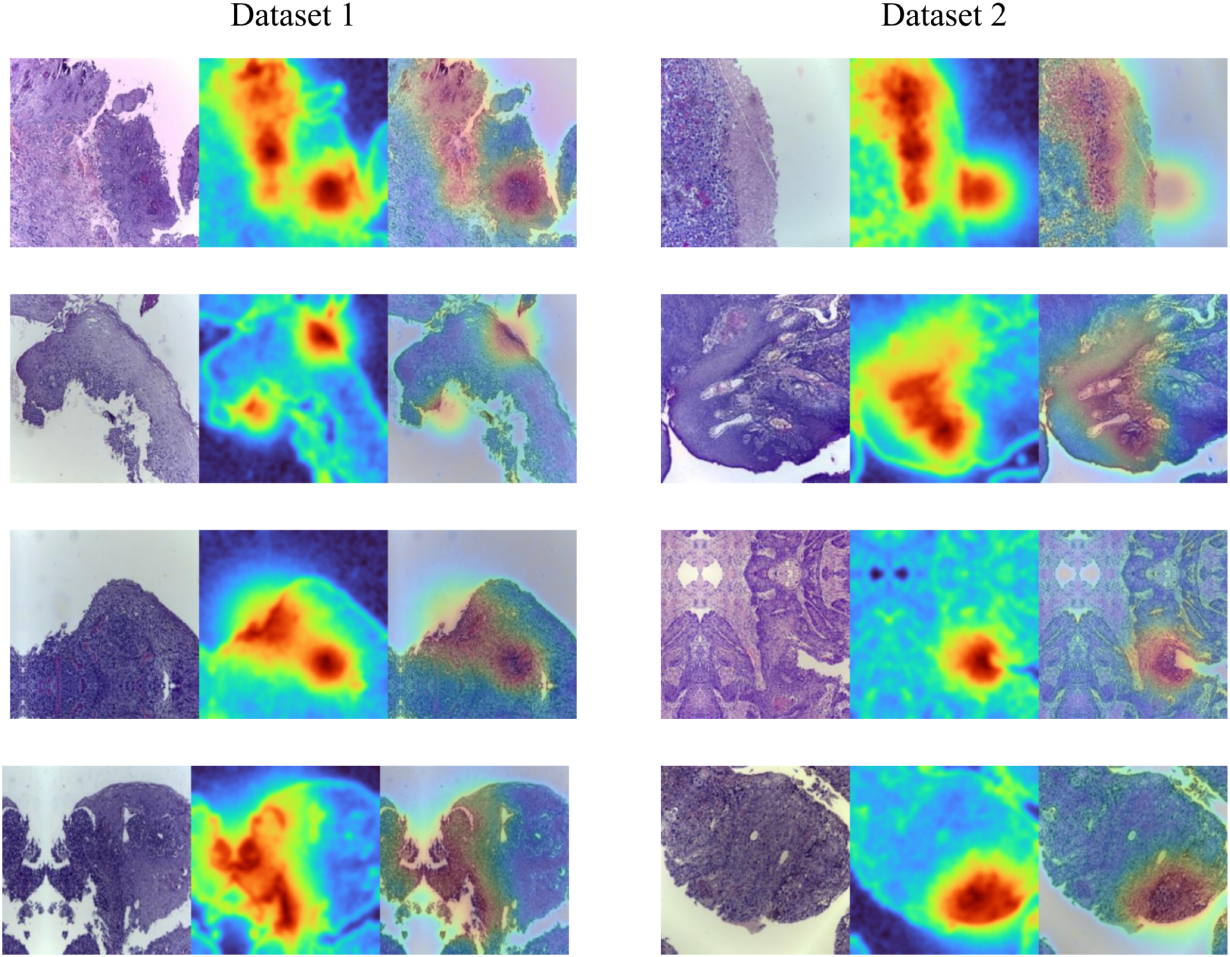
Grad-Cam experimental results.

To improve the interpretability of the model’s decisions and to verify whether the attended regions are consistent with pathological discriminative cues, we further employ Grad-CAM to visualize the key responsive regions of the network. This visualization reveals, from a spatial perspective, the local structures and tissue morphological evidence that the model relies on for samples of different classes, thereby providing intuitive support for the subsequent mechanism analysis. The results are shown in **Figure 7**.

From the Grad-CAM visualizations, it can be observed that the model shows a more focused and histologically consistent attention pattern on discriminative regions across both datasets. The highlighted areas are mainly located in epithelial hyperplasia regions and in local areas with dense nuclei or disordered morphological structures, rather than being dominated by large background textures or overall tonal differences. This indicates that the model relies more on morphological cues, rather than non-robust factors such as staining intensity, for decision making. In light of the proposed morphology-dominant encoding with staining-bias suppression, this module suppresses color-related components and strengthens structural components after the backbone features, making the attention responses more likely to concentrate on regions with stable morphological patterns and reducing the interference of staining fluctuations across slides on the saliency maps. Meanwhile, the spatial Transformer classification head performs patch-level spatial structured modeling and aggregation, enabling the model to build consistent spatial associations among multiple local evidences and preventing the attention from sparsely drifting to diagnostically irrelevant borders or blank regions. Notably, on Dataset 2, which exhibits stronger staining variations and more complex tissue morphology, the hotspot distribution remains relatively stable, further demonstrating that the synergy of the two modules not only improves classification performance but also enhances the stability and pathological plausibility of the attended regions from an interpretability perspective.

### 5.5 Hyperparameter sensitivity experimental results

#### 5.5.1 Learning rate hyperparameter sensitivity experimental results

**Figure 8:**
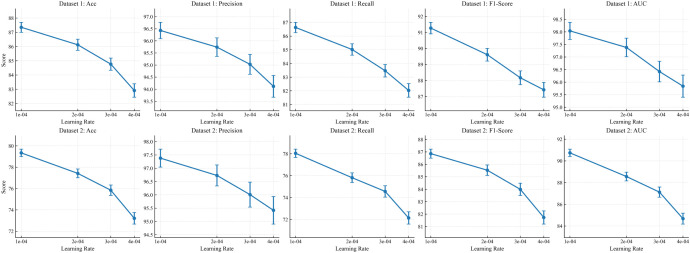
Learning rate sensitivity experiment results.

To evaluate the stability and reproducibility of the proposed method under perturbations of training hyperparameters, we further conduct a learning-rate sensitivity analysis. The learning rate directly determines the step size of parameter updates and the resulting optimization trajectory: an overly large value may cause oscillations or insufficient convergence, whereas an overly small value may reduce training efficiency and lead to suboptimal local minima. By systematically varying the learning rate while keeping all other settings unchanged, we can characterize the robustness of the model to optimization hyperparameters and provide guidance for selecting default configurations in practical applications. The results are reported in **Figure 8**.

From the learning-rate sensitivity curves, both datasets exhibit a consistent monotonic trend across different learning rates. As the learning rate increases from smaller values, metrics such as Acc, F1, and AUC generally decrease, and the fluctuation range becomes larger, indicating that the proposed method tends to fully leverage its structural modeling capability under more conservative update step sizes. From the perspective of the proposed designs, the morphology-dominant encoding with staining-bias suppression performs fine-grained reweighting and gated decomposition on backbone features. An overly large learning rate may cause the gating weights and the decomposed subspaces to drift too rapidly during early updates, which can reintroduce color-correlated noise into the representation and weaken the morphology-dominant advantage. Meanwhile, the spatial Transformer classification head needs to learn stable contextual dependencies over cross-patch spatial relations, and a larger step size can lead to oscillatory attention distributions, making structured aggregation less likely to form consistent selections of key regions, and ultimately resulting in a synchronized decline of overall metrics. Notably, this trend is more pronounced on Dataset 2, which has stronger staining variations and a larger scale, suggesting that when cross-domain shifts are more severe, the gains brought by morphology-robust representations and spatial aggregation rely more on a smooth optimization trajectory. Therefore, choosing a smaller learning rate can more reliably unleash the synergistic effects of the two modules and improve training controllability.

#### 5.5.2 Experimental results on the hyperparameter sensitivity of the optimizer

To further evaluate the robustness of the proposed method to changes in optimizer settings, we conduct a systematic sensitivity analysis on optimizer-related hyperparameters while keeping the network architecture and data splits unchanged. This experiment aims to examine how different configurations, such as momentum coefficients and weight decay, affect the stability of the optimization trajectory and the generalization behavior, thereby assessing the reproducibility of the method under realistic variations in training conditions. The comparative results also provide evidence for determining default training configurations and for interpreting the optimization characteristics of key modules. The results are reported in **Table 6**.

**Table 6:** Optimizer sensitivity results on the two datasets.

Dataset 1					
Optimizer	Acc	Precision	Recall	F1-score	AUC
AdaGrad	81.42 ± 0.463	93.14 ± 0.452	80.25 ± 0.498	86.11 ± 0.472	94.83 ± 0.451
SGD	83.57 ± 0.428	94.82 ± 0.417	82.44 ± 0.461	87.96 ± 0.439	95.71 ± 0.422
Adam	85.91 ± 0.392	95.63 ± 0.384	84.17 ± 0.429	89.47 ± 0.403	96.82 ± 0.387
RMSProp	84.74 ± 0.411	95.22 ± 0.396	83.52 ± 0.452	88.71 ± 0.431	96.24 ± 0.405
AdamW	87.35 ± 0.351	96.43 ± 0.338	86.63 ± 0.379	91.27 ± 0.361	98.04 ± 0.342

Dataset 2					
Optimizer	Acc	Precision	Recall	F1-score	AUC
AdaGrad	72.81 ± 0.512	95.24 ± 0.498	71.65 ± 0.538	81.42 ± 0.519	82.31 ± 0.503
SGD	74.36 ± 0.481	96.03 ± 0.452	73.12 ± 0.507	82.89 ± 0.489	84.54 ± 0.466
Adam	77.12 ± 0.412	96.92 ± 0.397	75.41 ± 0.446	85.42 ± 0.431	88.11 ± 0.402
RMSProp	75.91 ± 0.438	96.51 ± 0.421	74.36 ± 0.472	84.74 ± 0.451	86.97 ± 0.429
AdamW	79.34 ± 0.351	97.38 ± 0.338	78.04 ± 0.379	86.86 ± 0.361	90.74 ± 0.342

From the optimizer sensitivity comparisons, our method shows a consistent preference on both datasets for adaptive first-order optimization and decoupled weight decay. AdamW achieves the best Acc, F1, and AUC with relatively smaller variance, indicating that the training process relies more on stable update directions and appropriate constraints on parameter norms. From the perspective of the proposed innovations, the morphology-dominant encoding with staining-bias suppression involves feature decomposition and gated reweighting. When using strategies such as AdaGrad or plain SGD, it is easier to encounter imbalanced effective learning rates across different parameter subspaces or oscillatory updates, which makes the suppression of stain-related noise by the gating weights less stable and prevents structural cues from consistently dominating. Although Adam and RMSProp provide adaptive step sizes, they may still suffer from parameter magnitude drift when explicit regularization is needed, causing the relative weights between the morphology and stain branches to fluctuate in the mid-training stage. In contrast, the decoupled weight decay in AdamW more effectively constrains the growth of feature projection and attention parameters, allowing the morphology-dominant robust representations to converge faster to a stable subspace and providing a more consistent input distribution for the spatial Transformer classification head to perform structured cross-patch aggregation. As a result, AdamW maintains stronger overall discriminative capability and more reliable generalization on both datasets, especially on Dataset 2 where staining variations are more pronounced.

### 5.6 External validation experiments

In addition to the above internal evaluations on public datasets, we further design an external validation experiment to verify whether the proposed method can maintain stable diagnostic discrimination when applied to data collected under different real-world clinical workflows. Specifically, this experiment serves as an out-of-distribution assessment that focuses on robustness to variations in acquisition conditions and staining characteristics, and it is conducted in a strictly hold-out manner, where the external cohort is never used for any stage of model development. The corresponding ethics approval has been obtained in advance for this retrospective study, and the informed-consent requirement is waived according to applicable local laws and institutional regulations. The experimental results are shown in **Table 7**.

**Table 7:** External validation results when transferring models trained on public datasets to the external validation cohort.

Training → Testing	Acc (%)	Precision (%)	Recall (%)	F1-score (%)	AUC (%)
Dataset1 → External validation dataset	79.21	80.34	77.92	79.11	85.67
Dataset2 → External validation dataset	76.54	77.20	75.63	76.41	83.12

Overall, when models trained on the two public datasets are directly transferred to the external validation cohort, the classification metrics remain within a relatively stable range, indicating that the proposed method does not overly rely on dataset-specific imaging or staining patterns. This suggests that the method retains practical discriminative capability under cross-institutional and cross-acquisition conditions. The observation also provides indirect support for the robustness-oriented representation learning objective emphasized in our design, namely mitigating performance degradation induced by domain shift and maintaining consistent recognition behavior under real-world clinical distributions.

A comparison between the two training sources further shows that the model trained on Dataset1 exhibits a slightly better overall transfer performance than the one trained on Dataset2. This difference is commonly attributable to two factors: (i) the degree of similarity between the source domain and the external cohort in terms of acquisition workflow, magnification level, slide quality, and staining style; and (ii) discrepancies in class composition and noise characteristics across datasets, which may lead to different extents of bias in the learned decision boundary. Therefore, these results highlight that external generalization is jointly influenced by source–target domain alignment and the bias/noise structure within the source domain, and they also imply that more systematic cross-domain adaptation or multi-source training could further improve the stability on external cohorts.

### 5.7 Comparison of class imbalance methods

To mitigate the bias induced by the imbalanced distribution between normal and OSCC samples, we evaluate several widely used class-imbalance handling strategies within the same training pipeline. Specifically, we compare data-level resampling approaches, including SMOTE and random undersampling, with a loss-level reweighting approach based on Focal Loss. The following analysis reports the comparative performance of these methods under identical data splits and hyperparameter settings. The experimental results are shown in **Table 8**.

**Table 8:** Comparison of class imbalance handling methods on two datasets.

Dataset 1					
Method	Acc	Precision	Recall	F1-Score	AUC
SMOTE	84.12 ± 0.372	93.10 ± 0.361	83.40 ± 0.389	87.95 ± 0.374	95.62 ± 0.356
Undersampling	82.76 ± 0.401	91.85 ± 0.379	81.10 ± 0.416	86.14 ± 0.398	94.10 ± 0.382
Focal Loss	85.90 ± 0.363	94.70 ± 0.344	84.95 ± 0.372	89.56 ± 0.357	96.88 ± 0.341
Ours	87.35 ± 0.351	96.43 ± 0.338	86.63 ± 0.379	91.27 ± 0.361	98.04 ± 0.342

Dataset 2					
Method	Acc	Precision	Recall	F1-Score	AUC
SMOTE	76.20 ± 0.412	95.10 ± 0.367	74.60 ± 0.428	83.92 ± 0.401	87.30 ± 0.390
Undersampling	74.85 ± 0.438	93.60 ± 0.382	73.10 ± 0.446	82.06 ± 0.417	85.95 ± 0.402
Focal Loss	77.90 ± 0.398	96.40 ± 0.352	76.05 ± 0.410	85.45 ± 0.389	89.10 ± 0.371
Ours	79.34 ± 0.351	97.38 ± 0.338	78.04 ± 0.379	86.86 ± 0.361	90.74 ± 0.342

The comparative results on the two datasets indicate that relying solely on data-level resampling (SMOTE or random undersampling) can partially mitigate class skew, but the overall recall and decision stability remain limited; in particular, undersampling leads to more pronounced performance degradation due to the loss of majority-class information. In contrast, Focal Loss yields more balanced improvements across both datasets, suggesting that emphasizing hard examples at the loss level helps enhance minority-class recognition. Our strategy, which adopts stratified sampling and class-weighted cross-entropy loss, achieves the best performance on both datasets with consistent gains in Acc, Precision, Recall, F1, and AUC, demonstrating that it more effectively balances gradient contributions during training without introducing synthetic samples or sacrificing the majority-class distribution, thereby providing stronger minority coverage and more stable overall discrimination.

### 5.8 The impact of data volume on experimental results

**Figure 9:**
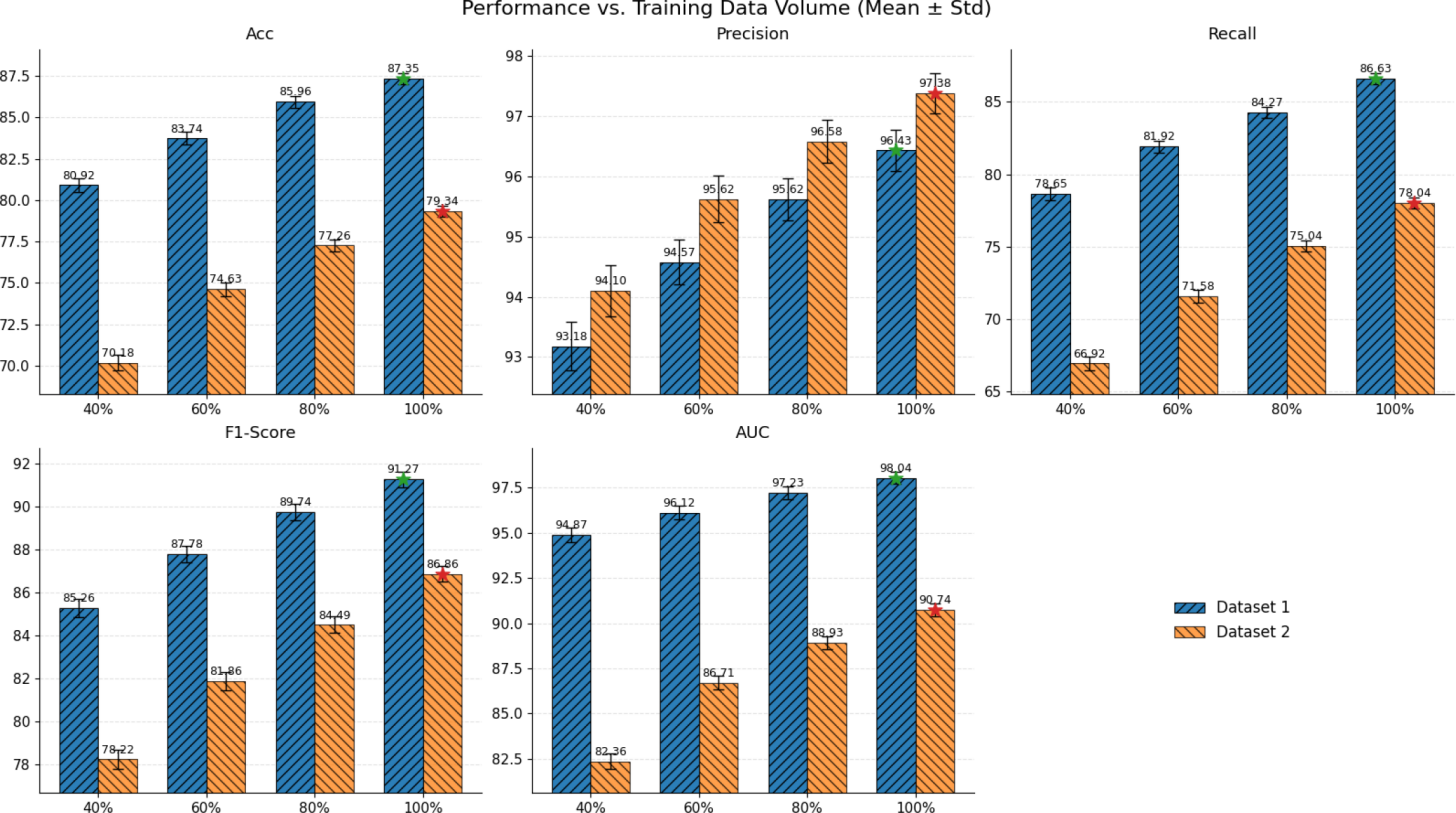
The impact of training data availability on experimental results.

To examine how training data availability influences model learning and generalization, we conduct a controlled data-volume study by progressively varying the proportion of training samples, preprocessing, and optimization settings unchanged. This setting allows us to isolate the effect of sample size on performance and to assess whether the proposed method remains stable under low-data regimes. The experimental results are shown in **Figure 9**.

From the figure, it can be observed that as the amount of available training data increases, all metrics on both datasets exhibit a consistent upward trend, indicating that the model can learn more stable and discriminative morphological and spatial-structural representations from a larger sample set. When the training data are limited, the performance fluctuations are more evident, whereas the variability gradually diminishes as the data scale grows, reflecting improved stability in both training dynamics and final predictions. Meanwhile, the two datasets follow broadly similar trends but differ in their overall performance levels, suggesting that data source characteristics and distribution complexity can influence learnability and the attainable performance ceiling. Overall, these results confirm that the proposed method scales favorably with data volume, remains effective under low-data regimes, and continues to gain more reliable generalization benefits when more data are available.

## 6 Limitation

One limitation of this study lies in the interpretability evaluation. Although we provide Grad-CAM visualizations to offer an intuitive, *post-hoc* indication of which regions contribute most to the classification decision, our task setting is image-level binary classification rather than semantic segmentation, and the used datasets only provide slide/image-level labels without pixel-level or ROI-level ground-truth annotations. Therefore, Grad-CAM is employed as a coarse localization tool for transparency and error analysis, rather than a lesion delineation mechanism, and the highlighted regions should not be interpreted as precise pathological boundaries. Under this weakly supervised setting, we cannot objectively quantify the overlap between attention regions and expert-defined diagnostic targets, which prevents reporting annotation-based interpretability metrics such as IoU/Dice or similarity coefficients.

In future work, we plan to strengthen interpretability validation by incorporating expert ROI annotations and multi-rater assessments. Specifically, we will collect or leverage datasets with pathologist-defined ROIs to enable quantitative agreement evaluation between model attention regions and expert annotations using IoU/Dice and related overlap measures. We will also consider multi-pathologist annotation protocols to estimate inter-observer consistency and to evaluate whether the model’s attention patterns align with expert consensus. In addition, we will explore weakly supervised localization and MIL-based region mining strategies to better bridge image-level supervision and region-level interpretability, thereby improving the clinical credibility and usability of the proposed framework.

## 7 Conclusion

This paper addresses the binary classification task of oral squamous cell carcinoma (OSCC) histopathology images and proposes an end-to-end classification framework that integrates staining-bias suppression and structured spatial aggregation. Specifically, we introduce a morphology-dominant encoding module with staining-bias suppression after the backbone features, which explicitly suppresses color-related components and strengthens structural components, so that the learned representations focus more on stable morphological cues. In addition, we design a Spatial Transformer Classifier Head that models spatial topological relations at the patch level and performs structured aggregation, thereby improving the integration of local diagnostic evidence. Systematic experiments on two public datasets demonstrate that the proposed method achieves stronger discriminative performance and more stable optimization behavior across comprehensive comparisons with multiple baselines, ablation studies, visualization-based validation, and hyperparameter sensitivity evaluations. Furthermore, Grad-CAM, confusion matrices, and feature distribution visualizations consistently support that the attended regions are aligned with histological discriminative cues.

Although the proposed method yields stable gains under different dataset scales and staining variations, cross-dataset transfer experiments still reveal performance degradation under significant distribution shifts, indicating that relying only on source-domain supervision is insufficient to fully cover imaging differences and sampling biases in real clinical settings. Future work will proceed along three directions. First, we will incorporate unsupervised or weakly supervised domain adaptation mechanisms to further reduce cross-domain gaps without increasing annotation costs in the target domain. Second, we will combine multi-scale context modeling with finer-grained spatial topological constraints to strengthen the joint modeling of large tissue structures and local cellular morphology. Third, we will conduct deeper validation on interpretability and clinical usability, for example, by quantifying attention consistency using key regions annotated by pathologists and exploring extended tasks related to grading or prognosis, so as to enhance the practical value and generalizability of the model in real-world scenarios.

## Data Availability

The original contributions presented in the study are included in the article/supplementary material. Further inquiries can be directed to the corresponding author.

## References

[1] Tan Y Wang Z Xu M Li B Huang Z Qin S . Oral squamous cell carcinomas: State of the field and emerging directions. Int J Oral Sci. (2023) 15:44. doi: 10.1038/s41368-023-00249-w. PMID: 37736748 PMC10517027

[2] Speight PM Abram TJ Floriano PN James R Vick J Thornhill MH . Interobserver agreement in dysplasia grading: Toward an enhanced gold standard for clinical pathology trials. Oral Surg Oral Med Oral Pathol Oral Radiol. (2015) 120:474–82. doi: 10.1016/j.oooo.2015.05.023. PMID: 26216170 PMC4564355

[3] Zhou Y Lu Y . Deep hierarchical multiple instance learning for whole slide image classification. In: 2022 IEEE 19th international symposium on biomedical imaging (ISBI). New York: IEEE (2022). p. 1–4.

[4] Van der Laak J Litjens G Ciompi F . Deep learning in histopathology: The path to the clinic. Nat Med. (2021) 27:775–84. doi: 10.1038/s41591-021-01343-4. PMID: 33990804

[5] Zhou Y Lu Y . Multiple instance learning with task-specific multi-level features for weakly annotated histopathological image classification. In: ICASSP 2022–2022 IEEE international conference on acoustics, speech and signal processing. New York: IEEE (2022). p. 1366–70.

[6] Tellez D Litjens G Bándi P Bulten W Bokhorst J-M Ciompi F . Quantifying the effects of data augmentation and stain color normalization in convolutional neural networks for computational pathology. Med Image Anal. (2019) 58:101544. doi: 10.1016/j.media.2019.101544. PMID: 31466046

[7] Gadermayr M Tschuchnig M . Multiple instance learning for digital pathology: A review of the state-of-the-art, limitations & future potential. Comput Med Imaging Graphics. (2024) 112:102337. doi: 10.1016/j.compmedimag.2024.102337. PMID: 38228020

[8] Yang S Li S Liu J Sun X Cen Y Ren R . Histopathology-based diagnosis of oral squamous cell carcinoma using deep learning. J Dent Res. (2022) 101:1321–7. doi: 10.1177/00220345221089858. PMID: 35446176

[9] Sukegawa S Ono S Tanaka F Inoue Y Hara T Yoshii K . Effectiveness of deep learning classifiers in histopathological diagnosis of oral squamous cell carcinoma by pathologists. Sci Rep. (2023) 13:11676. doi: 10.1038/s41598-023-38343-y. PMID: 37468501 PMC10356919

[10] Panigrahi S Nanda BS Bhuyan R Kumar K Ghosh S Swarnkar T . Classifying histopathological images of oral squamous cell carcinoma using deep transfer learning. Heliyon. (2023) 9:e13444. doi: 10.1016/j.heliyon.2023.e13444. PMID: 37101475 PMC10123069

[11] Ahmad M Irfan MA Sadique U Haq I Jan A Khattak MI . Multi-method analysis of histopathological image for early diagnosis of oral squamous cell carcinoma using deep learning and hybrid techniques. Cancers. (2023) 15:5247. doi: 10.3390/cancers15215247. PMID: 37958422 PMC10650156

[12] Das N Hussain E Mahanta LB . Automated classification of cells into multiple classes in epithelial tissue of oral squamous cell carcinoma using transfer learning and convolutional neural network. Neural Networks. (2020) 128:47–60. doi: 10.1016/j.neunet.2020.05.003. PMID: 32416467

[13] Das M Dash R Mishra SK . Automatic detection of oral squamous cell carcinoma from histopathological images of oral mucosa using deep convolutional neural network. Int J Environ Res Public Health. (2023) 20:2131. doi: 10.3390/ijerph20032131. PMID: 36767498 PMC9915186

[14] Koriakina N Sladoje N Bašić V Lindblad J . Deep multiple instance learning versus conventional deep single instance learning for interpretable oral cancer detection. PloS One. (2024) 19:e0302169. doi: 10.1371/journal.pone.0302169. PMID: 38687694 PMC11060593

[15] Zhou J Wu H Hong X Huang Y Jia B Lu J . A pathology-based diagnosis and prognosis intelligent system for oral squamous cell carcinoma using semi-supervised learning. Expert Syst Appl. (2024) 254:124242. doi: 10.1016/j.eswa.2024.124242. PMID: 41903563

[16] Lian W Lindblad J Stark CR Hirsch J-M Sladoje N . Let it shine: Autofluorescence of papanicolaou-stain improves ai-based cytological oral cancer detection. Comput Biol Med. (2025) 185:109498. doi: 10.1016/j.compbiomed.2024.109498. PMID: 39662319

[17] Wu W Xu X Gao C Diao X Li S Gui J . (2026). Exploiting label-independent regularization from spatial patterns for whole slide image analysis, in: Proceedings of the IEEE/CVF Winter Conference on Applications of Computer Vision (WACV), Los Alamitos: IEEE Computer Society. pp. 8639–49.

[18] Zheng Y Gindra RH Green EJ Burks EJ Betke M Beane JE . A graph-transformer for whole slide image classification. IEEE Trans Med Imaging. (2022) 41:3003–15. doi: 10.1109/tmi.2022.3176598. PMID: 35594209 PMC9670036

[19] Ding S Li J Wang J Ying S Shi J . Multi-scale efficient graph-transformer for whole slide image classification. IEEE J BioMed Health Inf. (2023) 27:5926–36. doi: 10.1109/jbhi.2023.3317067. PMID: 37725722

[20] Khan AM Rajpoot N Treanor D Magee D . A nonlinear mapping approach to stain normalization in digital histopathology images using image-specific color deconvolution. IEEE Trans BioMed Eng. (2014) 61:1729–38. doi: 10.1109/tbme.2014.2303294. PMID: 24845283

[21] Vahadane A Peng T Sethi A Albarqouni S Wang L Baust M . Structure-preserving color normalization and sparse stain separation for histological images. IEEE Trans Med Imaging. (2016) 35:1962–71. doi: 10.1109/tmi.2016.2529665. PMID: 27164577

[22] Janowczyk A Basavanhally A Madabhushi A . Stain normalization using sparse autoencoders (stanosa): Application to digital pathology. Comput Med Imaging Graphics. (2017) 57:50–61. doi: 10.1016/j.compmedimag.2016.05.003. PMID: 27373749 PMC5112159

[23] Zhou Y Wang H Bai Y Wan Y Jin C Chen M . Digital pathology image deblurring via local focus quality assessment. In: ICASSP 2024–2024 IEEE international conference on acoustics, speech and signal processing. New York: IEEE (2024). p. 2165–9.

[24] BenTaieb A Hamarneh G . Adversarial stain transfer for histopathology image analysis. IEEE Trans Med Imaging. (2017) 37:792–802. doi: 10.1109/tmi.2017.2781228. PMID: 29533895

[25] Lafarge MW Pluim JP Eppenhof KA Veta M . Learning domain-invariant representations of histological images. Front Med. (2019) 6:162. doi: 10.3389/fmed.2019.00162. PMID: 31380377 PMC6646468

[26] Anghel A Stanisavljevic M Andani S Papandreou N Rüschoff JH Wild P . A high-performance system for robust stain normalization of whole-slide images in histopathology. Front Med. (2019) 6:193. doi: 10.3389/fmed.2019.00193. PMID: 31632974 PMC6778842

[27] Otálora S Atzori M Andrearczyk V Khan A Müller H . Staining invariant features for improving generalization of deep convolutional neural networks in computational pathology. Front Bioeng Biotechnol. (2019) 7:198. 31508414 10.3389/fbioe.2019.00198 PMC6716536

[28] Salvi M Michielli N Molinari F . Stain color adaptive normalization (scan) algorithm: Separation and standardization of histological stains in digital pathology. Comput Methods Programs BioMed. (2020) 193:105506. doi: 10.1016/j.cmpb.2020.105506. PMID: 32353672

[29] Kang H Luo D Feng W Zeng S Quan T Hu J . Stainnet: A fast and robust stain normalization network. Front Med. (2021) 8:746307. doi: 10.3389/fmed.2021.746307. PMID: 34805215 PMC8602577

[30] Voon W Hum YC Tee YK Yap W-S Nisar H Mokayed H . Evaluating the effectiveness of stain normalization techniques in automated grading of invasive ductal carcinoma histopathological images. Sci Rep. (2023) 13:20518. doi: 10.1038/s41598-023-46619-6. PMID: 37993544 PMC10665422

[31] Zhou Y Che S Lu F Liu S Yan Z Wei J . Iterative multiple instance learning for weakly annotated whole slide image classification. Phys Med Biol. (2023) 68:155007. doi: 10.1088/1361-6560/acde3f. PMID: 37311470

[32] Zhou Y Lu Y . Multiple instance learning with critical instance for whole slide image classification. In: 2023 IEEE 20th international symposium on biomedical imaging (ISBI). New York: IEEE (2023). p. 1–5.

[33] Rahman TY Mahanta LB Das AK Sarma JD . Histopathological imaging database for oral cancer analysis. Data Brief. (2020) 29:105114. doi: 10.1016/j.dib.2020.105114. PMID: 32021884 PMC6994517

[34] Chaudhary N Rai A Rao AM Faizan MI Augustine J Chaurasia A . High-resolution ai image dataset for diagnosing oral submucous fibrosis and squamous cell carcinoma. Sci Data. (2024) 11:1050. doi: 10.1038/s41597-024-03836-6. PMID: 39333529 PMC11436638

[35] Chen T . Xgboost: A scalable tree boosting system. New York: Cornell University (2016).

[36] Loh W-Y . Classification and regression trees. Wiley Interdiscip Rev: Data Min Knowl Discov. (2011) 1:14–23. doi: 10.1002/widm.8. PMID: 22523608 PMC3329156

[37] He K Zhang X Ren S Sun J . Deep residual learning for image recognition, in: Proceedings of the IEEE conference on computer vision and pattern recognition, (2016). Los Alamitos: IEEE Computer Society. pp. 770–8.

[38] Simonyan K Zisserman A . Very deep convolutional networks for large-scale image recognition. (2014), arXiv preprint arXiv:1409.1556.

[39] Xie S Girshick R Dollár P Tu Z He K . (2017). Aggregated residual transformations for deep neural networks, in: Proceedings of the IEEE conference on computer vision and pattern recognition, Los Alamitos: IEEE Computer Society. pp. 1492–500.

[40] Liu Z Mao H Wu C-Y Feichtenhofer C Darrell T Xie S . (2022). A convnet for the 2020s, in: Proceedings of the IEEE/CVF conference on computer vision and pattern recognition, Los Alamitos: IEEE Computer Society. pp. 11976–86.

[41] Woo S Debnath S Hu R Chen X Liu Z Kweon IS . (2023). Convnext v2: Co-designing and scaling convnets with masked autoencoders, in: Proceedings of the IEEE/CVF conference on computer vision and pattern recognition, Los Alamitos: IEEE Computer Society. pp. 16133–42.

[42] Dosovitskiy A . An image is worth 16x16 words: Transformers for image recognition at scale. (2020), arXiv preprint arXiv:2010.11929.

[43] Sheakh MA Azam S Tahosin MS Karim A Montaha S Fahim KU . Ecgmlp: A novel gated mlp model for enhanced endometrial cancer diagnosis. Comput Methods Programs Biomed Update. (2025) 7:100181. doi: 10.1016/j.cmpbup.2025.100181. PMID: 41903563

[44] Tursun P Li S Li M Lv X Chen C Chen C . Dra-cn: A novel dual-resolution attention capsule network for histopathology image classification. In: Chinese conference on pattern recognition and computer vision (PRCV). Singapore: Springer (2024). p. 209–22.

[45] Xu Y Hong Y Li X Hu M . Medtrans: Intelligent computing for medical diagnosis using multiscale cross-attention vision transformer. IEEE Access. (2024). 12:146575. doi: 10.1109/access.2024.3450121. PMID: 41116384

[46] Rather IH Kumar S . (2024). Clash: A contrastive learning approach for few-shot classification of histopathological images, in: Proceedings of Fifth Doctoral Symposium on Computational Intelligence, pp. 265–77. Singapore: Springer.

[47] Hussain T Shouno H Hussain A Hussain D Ismail M Mir TH . Effresnet-vit: A fusion-based convolutional and vision transformer model for explainable medical image classification. IEEE Access. (2025) 13:54040–68 doi: 10.1109/access.2025.3554184. PMID: 41116384

[48] Remigio AS . Incarmag: A convolutional neural network with multi-level autoregressive moving average graph convolutional processing framework for medical image classification. Neurocomputing. (2025) 617:129038. doi: 10.2139/ssrn.4944536. PMID: 40330906

[49] Han M Qu L Yang D Zhang X Wang X Zhang L . Mscpt: Few-shot whole slide image classification with multi-scale and context-focused prompt tuning. IEEE Trans Med Imaging. (2025) 44:3756–69. doi: 10.1109/tmi.2025.3564976. PMID: 40299734

[50] He A Wu Y Wang Z Li T Fu H . Dvpt: Dynamic visual prompt tuning of large pre-trained models for medical image analysis. Neural Networks. (2025) 185:107168. doi: 10.1016/j.neunet.2025.107168. PMID: 39827840

